# Infection mechanisms of *Rhizoctonia cerealis* in wheat

**DOI:** 10.1002/imt2.70174

**Published:** 2026-10-05

**Authors:** Yan Ren, Ti Liu, Heying Zhao, Qirun Qian, Lufei Cui, Zhipeng Sun, Wenkai Yang, Peng Guo, Sisheng Wang, Lingran Zhang, Congwei Sun, Aolin Jia, Ning Zhang, Feng Chen

**Affiliations:** ^1^ State Key Laboratory of High‐Efficiency Production of Wheat‐Maize Double Cropping, Agronomy College Henan Agricultural University Zhengzhou China

**Keywords:** GWAS analysis, *R. cerealis* genomes, secreted effector protein, sharp eyespot resistance genes, wheat

## Abstract

*Rhizoctonia cerealis* (*Rc*) as a major pathogen causing sharp eyespot (SE) seriously affects wheat production worldwide. We collected 1683 wheat plants exhibiting SE symptoms from 30 locations in China over 7 years. We next constructed high‐quality genome maps by *de novo* sequencing of five representative *Rc* isolates and established an atlas of genomic variation by resequencing 207 isolates. We identified a key effector, RcPL3, conferring *Rc* pathogenicity. RcPL3 increased the protein accumulation of histone deacetylase TaHDA9 in wheat cytoplasm to promote *Rc* infection. TaHDA9 inhibited the E3 ubiquitin ligase TaRHC1A through deacetylation. RcPL3 indirectly inhibited the accumulation of TaRHC1A by mediating TaHDA9. TaRHC1A promoted the degradation of TaRBOH3 synthesizing reactive oxygen species (ROS) to cause wheat SE resistance. We further discovered an antagonist HY‐19741 to prevent *Rc* infection. The high‐quality genome maps of *Rc* and a SE RcPL3‐TaHDA9‐TaRHC1A‐TaRBOH3 regulatory cascade will be valuable for SE prevention in wheat.

## Highlights

- We constructed high‐quality genome maps of five representative *Rc* isolates by de novo sequencing and established its atlas of genomic variation.
- We identified a key effector *RcPL3* conferring *Rc* pathogenicity and proposed a working model RcPL3‐TaHDA9‐TaRHC1A‐TaRBOH3 regulating SE resistance in wheat.
- We found out HY‐19741 as an antagonist to effectively impede *Rc* infection.

## Graphical abstract

We discover that *R. cereali*s (*Rc*) is a primary pathogen causing sharp eyespot of wheat after 335 distinct isolates were isolated from 1683 SE‐diseased wheat plants across China over 7 years using a single hyphal tip isolation method. We then generated high‐quality reference genomes via de novo sequencing of five representative *Rc* isolates and constructed a genomic variation atlas by resequencing 207 *Rc* isolates. We further identify *RcPL3* as a key effector by integrating genome, transcriptome, and genome‐wide association study analyses, and find that RcPL3 recruits TaHDA9 to suppress TaRHC1A by deacetylation and thus inhibits degradation of TaRBOH3 by TaRHC1A, finally resulting in ROS burst that contributed to SE susceptibility in wheat plants. Furthermore, we identified HY‐19741 as an antagonist to effectively impede *Rc* infection by weakening the interaction of RcPL3 and TaHDA9. This work reveals a novel fungal pathogenic cascade and provides a candidate agent for wheat disease control.

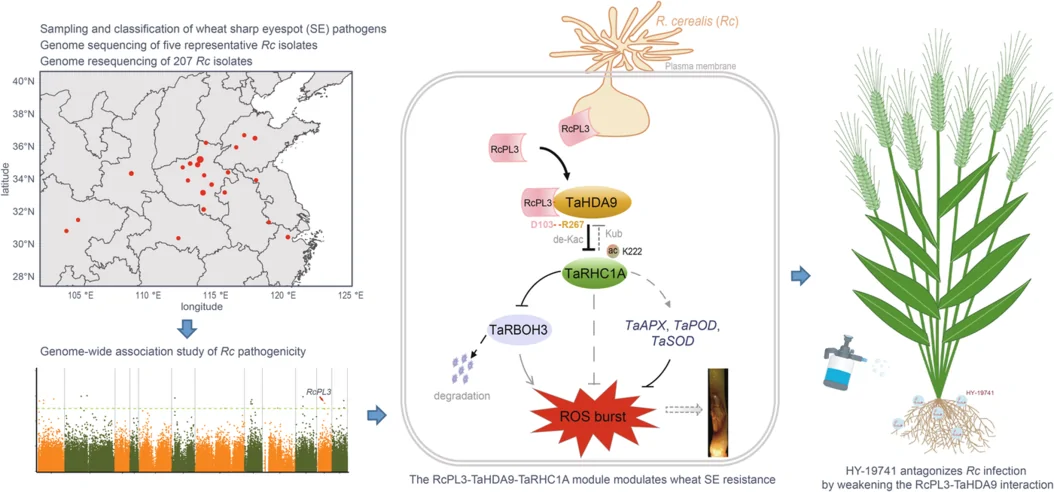

## INTRODUCTION

*Rhizoctonia*, a genus of ubiquitous and variable soil‐borne pathogenic fungi, causes diseases that affect many species of plants. The binucleate *R. cerealis* (*Rc*) causes not only a yellow patch of turfgrasses and root rot of sugar beet, cotton, potato, and several legumes, but also sharp eyespot (SE) in wheat. Therefore, *Rc* is a leading cause of serious economic losses in a range of crop plants [1]. As *Rc* does not form asexual spores, and its sexual stage is probably rare in nature [2], it survives between cropping cycles as vegetative mycelium and sclerotia in plant debris or soil [3]. In China, about 8 million hectares of wheat are affected by SE each year, and the yield from individual fields can be damaged by 10%–40% [1]. In Europe, the incidence of SE is increasing due to earlier sowing, planting of highly susceptible cultivars, and more favorable weather for infection.

Fungicides are commonly used to control SE, but usage is increasingly discouraged due to concerns about food contamination, effects on off‐target organisms, and risk of fungicide resistance [4, 5]. Host resistance against *Rc* is a more effective and environmentally safe alternative. However, no immune or highly SE‐resistant wheat germplasm has been identified [6, 7]. Accurate genomic information for plant pathogens and knowledge of their candidate effectors could allow us to unravel the mystery of the molecular mechanism of diseases during the infection of crops. In turn, this understanding is of crucial importance for the creation of resistant germplasm.

Genomic sequences of *R. solani* (*Rs*) isolates from anastomosis groups AG1‐1A, AG1‐1B, AG3, AG8, AG5, and so on have become available (https://www.ncbi.nlm.nih.gov/datasets/genome/), which are potentially useful for understanding host adaptation, host specificity, and host range of *Rs* [8, 9, 10, 11, 12]. Although the whole‐genome assembly and annotation of two highly virulent *Rc* isolates, Rc207 and R0301, were published recently [13, 14], only limited information is available on the genetic diversity and pathogenic mechanisms of *Rc*. In‐depth sequencing of multiple isolates and large‐scale resequencing of the *Rc* genome will facilitate the characterization of the *Rc* genome and genetic control of pathogenicity. The use of bacterial genomes has led to the identification of pathogenicity genes in *Escherichia coli*, *Helicobacter pylori*, and other bacterial species that colonize the human microbiome [15, 16].

The genomes of plant pathogens encode many putative effector proteins that play essential roles in pathogenicity [17, 18, 19, 20, 21]. For example, the invasive hyphae of *F. graminearum*, the causal agent of Fusarium head blight (FHB) on wheat and barley, secrete effector Osp24 that is required for infectious growth in wheat rachis tissues [19]. PsSpg1, a fungal effector necessary for *Puccinia striiformis f. sp. tritici* (*Pst*) pathogenicity, promotes parasitism in a TaPsIPK1‐dependent manner [20]. *Phytophthora* spp. effector PSR2 hijacks the Ser/Thr protein phosphatase 2A (PP2A) core enzyme in plant hosts to promote disease [21]. However, in comparison with other plant pathogens, the underlying mechanisms of how *Rc* effectors disrupt plant defense response remain unclear.

Proteins with post‐translational modifications (PTMs) can modulate diverse cellular processes, including responses to abiotic and biotic stresses in plants [22]. Primarily mediated by poly(ADP‐ribose) polymerase (PARP) proteins in *Arabidopsis* are activated and PARylated in response to infection by pathogens [23]. Activated PARPs recruit UBC13 (the ubiquitin conjugating enzyme that catalyzes the synthesis of K63‐linked polyubiquitin chains) and protein disulfide‐isomerases (PDIs) by physical interaction and PARylation, in turn facilitating K63‐linked ubiquitination of PDIs mediated by UBC13 to activate PDIs, thereby enhancing plant immunity [23]. In cotton, lysine 2‐hydroxyisobutyrylation (Khib) and succinylation (Ksuc) are strongly associated with resistance to *Verticillium dahlia* and genes *GhHDA15* and *GhSRT1* negatively regulate Verticillium wilt resistance by removing Khib and Ksuc [24]. In rice, phosphorylation of transcription factor OsWRKY31 by an OsMKK10‐2‐OsMPK module confers enhanced resistance to *M. oryzae* [25]. These examples indicate that PTMs can have negative and positive roles in regulating plant immune response. In addition, pathogen effectors can suppress plant immunity by targeting various types of PTM. The PsSpg1 effector from *Pst* interacts with wheat kinase TaPsIPK1, enhancing its kinase activity, whereas TaPsIPK1 phosphorylates TaCBF1d, thereby facilitating pathogen infection [20]. Another *Pst* effector, Hasp98, facilitates pathogenicity by blocking the kinase activity of wheat TaMAPK4 [26]. The *Magnaporthe oryzae* effector AvrPiz‐t targets the really interesting new gene (RING) E3 ubiquitin ligase APIP6 in rice to suppress basal defense [27]. The *F. graminearum* effector OSP24 interacts with SNF1‐related kinase TaSnRK1ɑ to stimulate its proteasomal degradation, which in turn negatively impacts resistance to fungal infection in wheat plants [19].

Wheat SE resistance is controlled by quantitative trait loci (QTL), and the effect of individual QTL is generally minor [28]. Therefore, it is very difficult to identify and clone a QTL/gene for resistance to SE by forward genetics. At present, no identified QTL has been used in wheat breeding, and there is no wheat cultivar with stably high resistance to SE. However, by analyzing effector proteins in *Rc* and understanding the mechanism of pathogenicity, it is possible to emerge a more efficient strategy in breeding for SE resistance. Therefore, we deciphered five high‐quality genomes of *Rc*, characterized genomic variation by resequencing, and further developed a *Rc* effector library. We also identified pathogenic effector RcPL3 that recruits histone deacetylase TaHDA9 for expediting infection and enhancing deacetylation and degradation of E3 ligase TaRHC1A by TaHDA9. Characterization of the *Rc* genome and the RcPL3‐TaHDA9‐TaRHC1A cascade regulating *Rc* infection will provide important information to help in breeding for resistance to this disease.

## RESULTS

### Collection and classification of sharp eyespot (SE) pathogens in the major wheat growing regions of China

Based on the typical visual symptom of sharp eyespot (SE), we collected 1683 SE‐diseased wheat plants from 30 locations covering almost all major wheat regions in China during the years 2014 to 2020 (Figure 1A,B). We made 335 isolates from the collected samples using a single hyphal tip isolation method. By sequencing the rDNA internal transcribed spacer (ITS) region, we classified these isolates as *R. cerealis* (267), *F. pseudograminearum* (13), *A. alternata* (9), *C. tenuissimum* (9), *C. cochliodes* (6), and others (Figure 1C). Fluorescence microscopy of all the 267 *Rc* isolates confirmed two nuclei per cell and hyphal septa characteristics (Figure S1A), supporting the ITS sequencing data. Regional distribution results showed that *Rc* was present in 22 of the 30 sampling locations, indicating that *Rc* was the main pathogen causing SE of wheat in China. Binucleate *Rhizoctonia* spp. were earlier divided into 21 anastomosis groups (AGs) (AG‐A to AG‐U), but only 17 AGs (Table S1) were confirmed due to misclassification, duplication, and isolate loss [29]. To classify our *Rc* isolates for AGs, we cloned and sequenced the ITS regions; 168 isolates possessed a single ITS sequence, whereas the remaining 99 had multiple ITS sequences (Table S1), suggesting that the multiple ITS sequences are common in *Rc*. Phylogenetic analysis revealed two distinct clades among the isolates; 79.8% were in AG‐D subgroup I (AG‐DI), whereas 20.2% possibly belonged to a new AG temporally designated AG‐DIV (Figure S1B).

**FIGURE 1:**
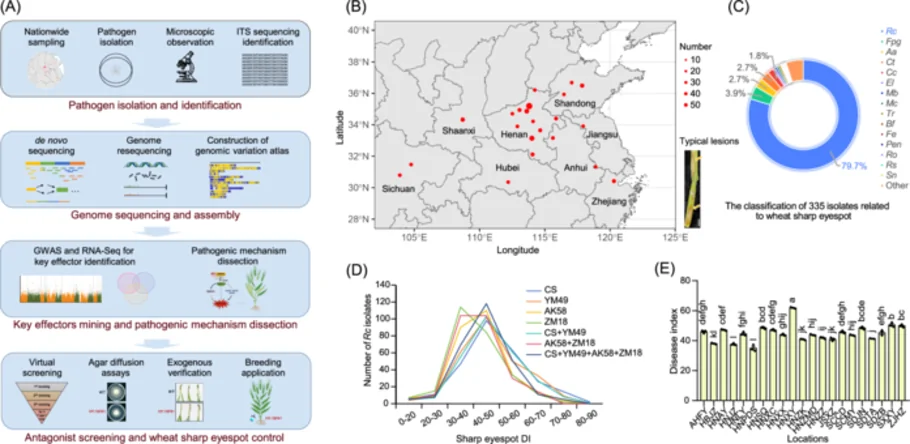
Collection sites and identification of pathogens in sharp eyespot (SE) samples from major wheat regions in China. (A) The experimental design and analytical procedure of this study. (B) Distribution of the 22 *Rhizoctonia cerealis* (*Rc*) sampling locations and SE symptoms. Red dot size represents the number of *Rc* isolates. Yellow arrows indicate typical SE lesions. Scale bar, 3 mm. This figure is based on the Standard Map Service of the Ministry of Natural Resources of the People's Republic of China (Map review No. GS(2024)0650). (C) Pathogens identified in all 335 isolates based on the rDNA internal transcribed spacer (ITS) sequence analysis. *Fpg*, *Fusarium pseudograminearum*; *Aa, Alternaria alternata*; *Ct, Cladosporium tenuissimum*; *Cc, Chaetomium cochliodes*; *El, Epicoccum layuense*; *Mc, Mucor circinelloides*; *Tr, Trichothecium roseum*; *Bf, Botrytis fabae*; *Fe, Fusarium equiseti*; *Mb, Microdochium bolleyi*; *Mn, Microdochium nivale*; *Pen, Penicillium* spp.; *Ro, Rhizopus oryzae*; *Rs, Rhizoctonia solani*; *Sn, Silene noctiflora*. (D) The frequency distribution of averaged disease index for 267 *Rc* isolates in seven *Rc* pathogenicity phenotypes. CS, Chinese Spring. YM49, Yumai 49. ZM18, Zhoumai 18. AK58, Aikang 58. DI, Disease index. (E) Variance analysis of the average disease index for *Rc* isolates from 21 sampling locations. Values are means ± SE of three biological replicates. Different letters indicate significant differences (*p* < 0.05), as determined by one‐way analysis of variance (ANOVA) with Tukey's multiple comparisons test. AHFY, Fuyang, Anhui Province; HBJZ, Jingzhou, Hubei Province; HNAY, Anyang, Henan Province; HNJZ, Jiaozuo, Henan Province; HNLY, Luoyang, Henan Province; HNPDS, Pingdingshan, Henan Province; HNSQ, Shangqiu, Henan Province; HNXC, Xuchang, Henan Province; HNXX, Xinxiang, Henan Province; HNXY, Xinyang, Henan Province; HNZK, Zhoukou, Henan Province; HNZMD, Zhumadian, Henan Province; HNZZ, Zhengzhou, Henan Province; JSXZ, Xuzhou, Jiangsu Province; SCCD, Chengdu, Sichuan Province; SCMY, Mianyang Sichuan Province; SDJN, Jinan Shandong Province; SDTA, Taian Shandong Province; SDZB, Zibo, Shandong Province; SXXY, Xianyang, Shaanxi; ZJHZ, Hangzhou, Zhejiang Province.

We identified the pathogenicity of all 267 *Rc* isolates using wheat cultivars Aikang 58 (AK58, averaged DI = 42.98), Zhoumai 18 (ZM18, averaged DI = 41.12), Chinese Spring (CS, averaged DI = 47.65), and Yumai 49 (YM49, averaged DI = 47.00) (Tables S1 and S2). All 267 *Rc* isolates caused typical SE symptoms (moire‐like lesions at the basal leaf sheaths), and the average disease index (DI) of the four cultivars of the above‐mentioned 267 *Rc* isolates ranged from 11.75 to 75.44 (Table S1 and Figure 1D). Among these isolates, 16 (including R0301) and 13 (including R1‐8 and R10‐1) generated DI of ≥60 and ≤30, respectively, implying their distinctly pathogenic differences. *Rc* isolates sampled from different locations showed significant differences in pathogenicity (Figure 1E), suggesting regionally based differences in pathogenicity.

### Genome sequencing and characterization of five *Rc* isolates

Five AG‐DI isolates comprising one highly pathogenic isolate (R0301), two moderately pathogenic isolates (R1‐6 and R5‐3), and two weakly pathogenic isolates (R1‐8 and R10‐1) were subjected to *de novo* genome sequencing using the Illumina and PacBio Sequel platforms. The Illumina short‐read sequencing yielded an average depth of 128.15× with the range of 119.27–143.34×. A total of 3.72 Gb circular consensus sequencing (CCS) reads were obtained across all five isolates, providing an average PacBio CCS coverage of 16.00×, ranging from 11.61× to 22.99× based on assembled genome sequences. The assembled genomes varied between 42.53 Mb and 49.34 Mb, with contig N50 values from 1.78 to 2.89 Mb (Table S3). Benchmarking universal single‐copy orthologs (BUSCO) analysis revealed that complete BUSCO values ranged from 275 (94.83%) to 283 (97.57%), 2 to 28 (0.69%–9.66%) of which were duplicated (Table S4). Three genomes (R0301, R5‐3, and R1‐8, which exhibit diverse pathogenicity) were further assembled at the chromosome level, with scaffold N50 sizes of 3.85–4.70 Mb by chromatin conformation capture (Hi‐C) sequencing. An averaged 93.75% of all assembled sequences was anchored on 12 chromosomes (Chr.1–Chr.12) (Table S3 and Figure 2A–D). Analysis of genome collinearity revealed high consistency between the assembled sequences of R0301, R1‐8, and R5‐3 (Figure 2E).

**FIGURE 2:**
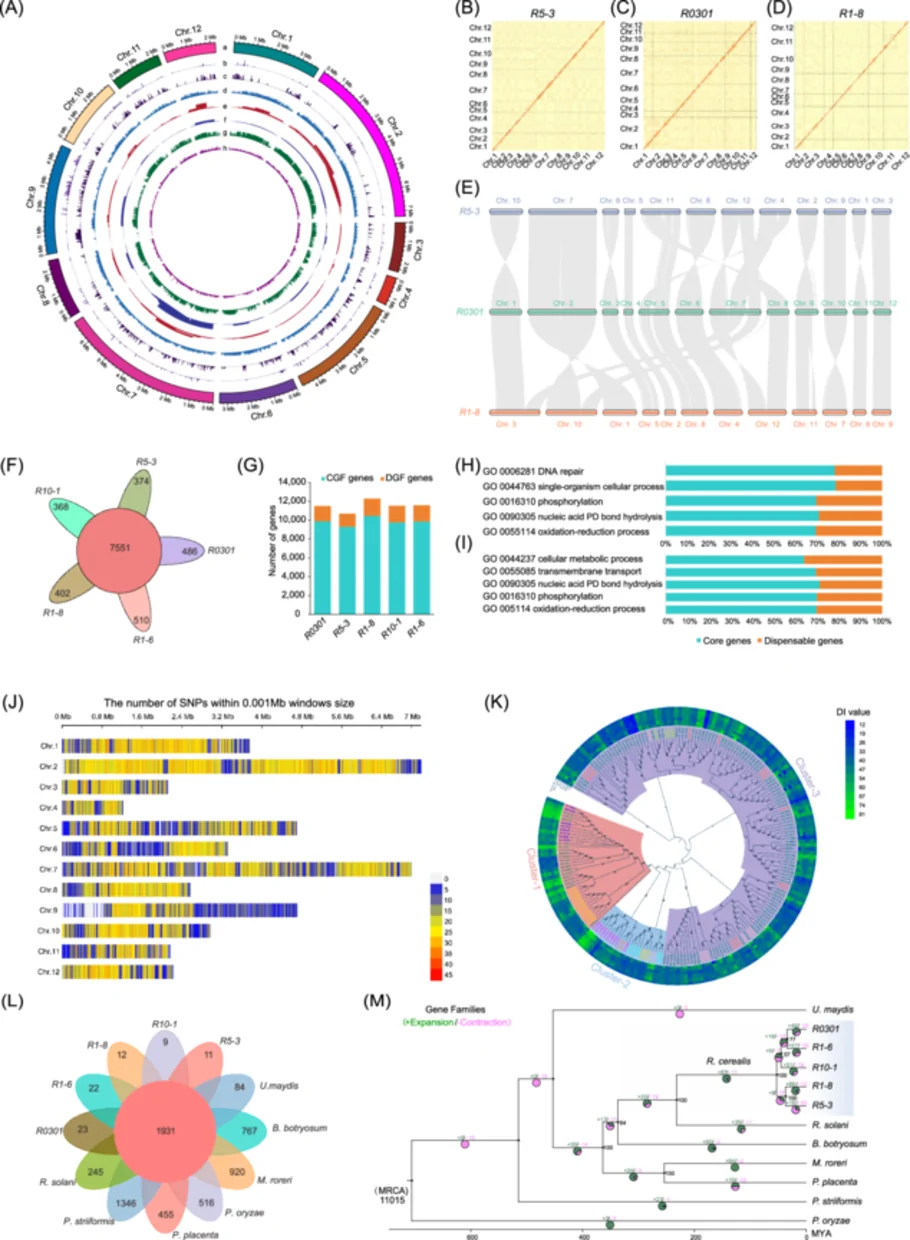
Characterization of *Rc* genomes, atlas of variation, and evolutionary analyses among Basidiomycete species. (A) Genomic features of *Rc* isolate R0301. a, The chromosome names and relative sizes. b and c, DNA transposon and retrotransposon density. d−f, Gene distribution, expression before infection, and 5 dpi (days post inoculation) with R0301. g and h, Distribution of SNPs and INDELs. (B−D) Interaction heatmaps of the Hi‐C assembled chromosomes in *Rc* isolates R5‐3 (B), R0301 (C), and R1‐8 (D). (E) Genomic collinearity among R0301, R5‐3, and R1‐8. (F) Flower plot display of core orthogroups (number in the center) and isolate‐specific orthogroups (numbers in the petals) for 5 *Rc* isolates (R0301, R5‐3, R1‐8, R10‐1, and R1‐6). (G) Numbers of CGF and DGF genes in each *Rc* isolate. (H and I) Top five GO terms enriched in the CGF (H) and DGF (I) gene sets. (J) Heatmap displaying SNP numbers on *Rc* chromosomes. (K) The phylogenetic tree by co‐analysis of genome variation and virulence traits. (L) Flower plot display of core orthogroups (number in the center) and species‐specific orthogroups (numbers in the petals) for the 12 fungal genomes. (M) Phylogenetic tree of *Rc* and seven other fungal genomes. The pie charts on branches indicate the number of genes in expanded(+)/contracted(−) gene families in fungal species. CGF, core gene family; DGF, dispensable gene family; DNA, deoxyribonucleic acid; GO, gene ontology; Hi‑C, high‐throughput chromosome conformation capture; INDELs, insertions‐deletions; SNPs, single‐nucleotide polymorphisms.

Analysis indicated that 24.29–26.34% of the five *Rc* genomes were repetitive elements (Table S3); among which long terminal repeat retrotransposons were the most abundant (13.70–15.42%) (Table S4). Prediction of protein‐coding genes indicated that each genome possessed 10,697–12,296 genes with average genic lengths of 2356–2405 bp and CDS lengths of 224–227 bp (Table S4). More than 96.48% of the predicted genes in each genome were matched by transcriptome‐based prediction and reference protein sequences from two other Basidiomycota genomes (Figure S1C). Additionally, we predicted 63–254 ribosomal RNAs, 132–224 transfer RNAs, and 43–100 other non‐coding RNAs in the five *Rc* genomes.

Ortholog clustering using OrthoFinder software [30] revealed 11,411 orthogroups (roughly comparable with gene families), including 7551 core gene families (CGF, present in all five *Rc* genomes) and 3860 dispensable gene families (DGF, absent in at least one *Rc* genome) (Figure 2F). Gene annotation in the Pfam database indicated that CGF mainly included protein kinase major facilitator superfamily, F‐box‐like, methyltransferase, and RNA recognition motifs, whereas the DGF mainly included DDE (a family of transposase proteins necessary for DNA transposition), superfamily endonucleases, multicopper oxidases and retrotransposon gag proteins (Figure S1D,E). Furthermore, when accumulating three of the five isolates, the additional gene family numbers contributed by each added isolate was declined significantly. After adding the fifth isolate in the pan‐genome, only 374 additional gene families were detected (Figure S1F,G). More than 84.71% of genes belonged to CGF in each *Rc* genome (Figure 2G and Table S4). Gene ontology (GO) analysis revealed that CGF genes were mainly enriched in oxidation‐reduction processes, nucleic acid phosphodiester (PD) bond hydrolysis, and phosphorylation (Figure 2H), whereas DGF genes were mainly enriched in transmembrane transport and cellular metabolism (Figure 2I).

### Construction of an atlas of genomic variation in *Rc* by resequencing genomes

Genome resequencing of 207 *Rc* isolates (Table S1) was undertaken by Illumina Hiseq to identify nucleotide variants within the *Rc* genome. After filtering for quality control, 256.51 Gb clean data were generated and mapped to the R0301 genome. Each genome had an average 24‐fold coverage depth, and the mapping rate varied from 84.89% to 95.19% among libraries. We finally obtained 719,791 high‐quality single nucleotide polymorphisms (SNP) (Figure 2J), with 15.72% (113,128) and 84.28% (606,663) located in intergenic and genic regions, respectively. The gene‐based SNPs covering ±5Kb of genes comprised 277,912 exonic SNPs, 65,992 intronic SNPs, 15,058 splice‐site SNPs, 21,441 UTR SNPs, 225,958 upstream/downstream SNPs, and 302 intragenic (no transcript information) SNPs.

The phylogenetic tree by co‐analysis of genome variation and pathogenicity indicated that *Rc* isolates in Cluster‐1 (mainly from Sichuan, Shaanxi, and Xinyang) exhibited a significantly stronger virulence than those in Cluster‐2 (mainly from Zhejiang and Hubei) and Cluster‐3 (mainly from Shandong, Jiangsu, Anhui, Henan excluding Xinyang) (Figures 2K and S1H), implying that the pathogenicity was closely associated with genome variation in *Rc* isolates.

### Evolution and comparative genomics with basidiomycete members

Ortholog clustering in the 5 *Rc* genomes and 7 available fungal species genomes (6 Basidiomycetes and 1 Ascomycete) from NCBI identified 15,435 orthogroups (gene families, GF) across the 12 genomes. Among them, 1931 GF (12.51%) were in all 12 genomes, and 77 containing 167 genes were *Rc*‐specific (Figure 2L and Table S5). However, all 167 *Rc‐*specific genes belonged to DGF in the *Rc* pan‐genome. A comparison among the 12 fungal genomes revealed that 87 and 11 GF underwent expansion and contraction events in the *Rc* genome, respectively (Figure 2M). Gene annotation in the Pfam database indicated that the expanded genes were mainly enriched in DDE superfamily endonucleases, methyltransferase domains, and protein kinase domains (Table S6). Phylogenetic analysis revealed that *Rc* is closest to *R. solani* (*Rs*) among the 6 Basidiomycete*s*. Analysis of genome collinearity also revealed that *Rc* and *Rs* had relatively high collinearity (Figure S2). Divergence between *Rc* and *Rs* was predicted to have occurred ~232 million years ago (MYA), whereas *Rhizoctonia* and the other five Basidiomycetes diverged from ~337 MYA (Figure 2M).

### Prediction of effector proteins in *Rc* pan‐genome

Plant‐pathogenic fungi secrete effector proteins that facilitate infection of the host species [20, 31, 32]. Effector proteins have a signal peptide but lack a transmembrane domain [13, 33]. Of the 3757 potential secreted proteins predicted in the five *Rc* genome by protein subcellular location analysis (Figure 3A,B), 506 were predicted to be effector proteins using both EffectorP 2.0 and KohGPI softwares (Figure 3A,B), and 98, 90, 102, 105, and 111 corresponding genes were identified in the sequenced genomes of R0301, R5‐3, R1‐8, R10‐1, and R1‐6, respectively. Ortholog clustering of *Rc* effector genes from five *Rc* isolates belonged to 105 GF, including 53 CGF and 52 DGF (Figure S3). Gene annotation in the Pfam database indicated that CGF mainly included pectate lyase and rare lipoprotein A (RlpA)‐like double‐psi beta‐barrel, whereas DGF mainly included beta/gamma crystallin and 3‐dehydroquinate synthase (Table S7). These effector genes are potential pathogenicity‐related genes in *Rc*.

**FIGURE 3:**
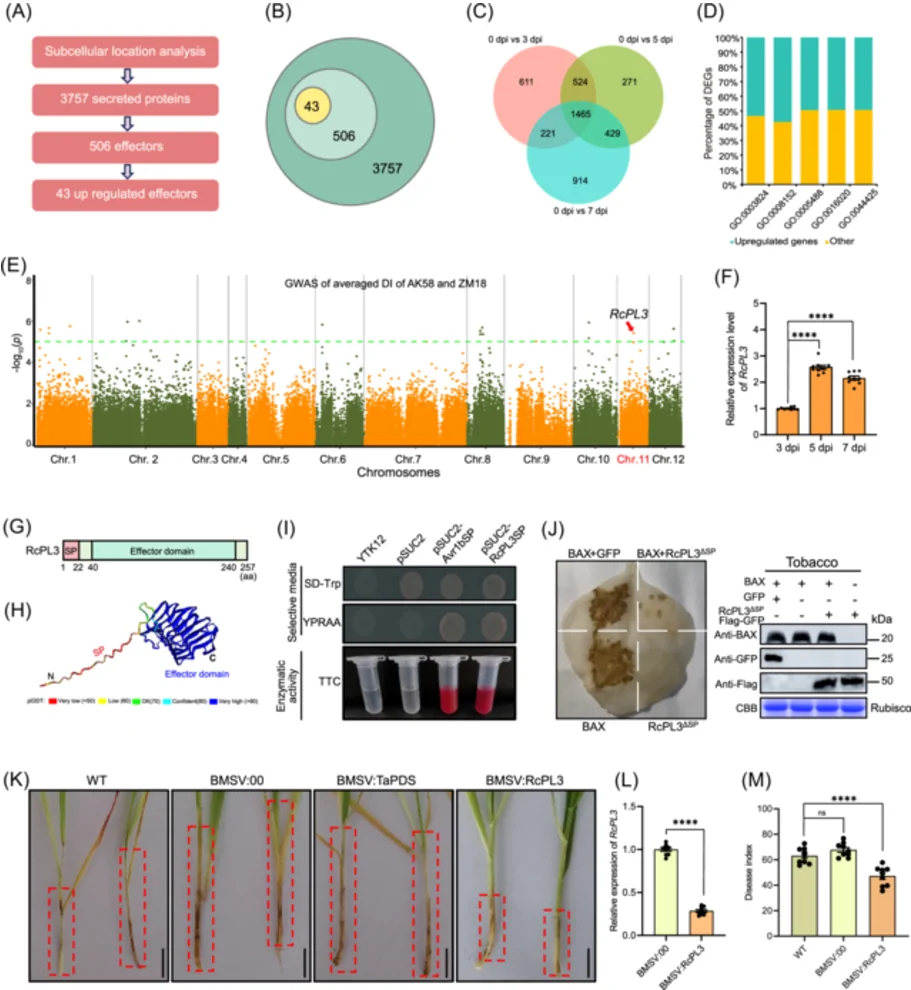
Identification and functional verification of effector protein RcPL3. (A) Prediction of subcellular protein locations identified 3757 potential secreted proteins in the five *Rc* isolates; 506 were predicted effector proteins; 43 up‐regulated effector genes were detected by RNA‐Seq during infection. (B) Venn diagram of 3757 potential secretory protein genes, 506 effector protein genes, and 43 up‐regulated effector genes. (C) Venn diagram of differentially expressed genes (DEGs) derived from pairwise comparisons (0 dpi vs. 3 dpi, 0 dpi vs. 5 dpi, and 0 dpi vs. 7 dpi). (D) Top five GO terms enriched in up‐regulated and down‐regulated genes. These terms include catalytic activity (GO:0003824), metabolic process (GO:0008152), binding (GO:0005488), membrane (GO:0016020), and membrane part (GO:0044425). (E) Manhattan plot of *Rc* pathogenicity identified by genome‑wide association study (GWAS). Red arrow represents the SNP locus for *RcPL3*. Green dashed lines indicate the genome‑wide significance threshold (*p* value = 1.0e‐5). (F) Expression levels of the *RcPL3* gene in infected wheat plants at 3, 5, or 7 dpi with R0301 by quantitative real‑time polymerase chain reaction (qRT‐PCR). Values are means ± SE of three biological replicates. (G) Protein structure of RcPL3. (H) Predicted three‐dimensional structure of the RcPL3 protein by Alphafold2 software. (I) The yeast suc2 mutant YTK12 and its transformants expressing the empty vector pSUC2 or vectors with the signal peptide from RcPL3 and Avr1b (positive control) were assayed for growth on SD‐Trp‐glucose agar or YPRAA plates and invertase activity in TTC medium. (J) Transient expression of *RcPL3* suppressed programmed cell death (PCD) triggered by Bcl‐2‐associated X protein (BAX) (left); Identification of the protein levels of BAX and RcPL3^ΔSP^ (right). At the indicated spots, *N. benthamiana* leaves are infiltrated with *Agrobacterium* cells expressing BAX/RcPL3^ΔSP^ alone or infiltrated with BAX expressing cells at 24 h after infiltration with GFP or RcPL3^ΔSP^ first. Representative leaves are photographed at 4−5 days after infiltration. (K−M) Virus‐induced gene silencing (VIGS) verifies the function of *RcPL3*. Phenotypes (K), relative expression levels (L), disease index (M) (12 plants per replicate, *n* = 9) of BSMV:00, BSMV:TaPDS, and BSMV:RcPL3 plants inoculated with *Rc* isolate R0301. The red dashed box in (K) shows the infected sites by *Rc*. Scale bars, 1 cm in (K). Data in (F and M) are presented as the mean values ± SE with individual data point (****p < 0.0001; one‐way ANOVA with Dunnett's multiple comparisons test). Data in (L) are presented as the mean values ± SE with individual data point (****p < 0.0001; unpaired two‐sided *t*‐test with at least three biologically independent replicates). In (I−K), the experiments were conducted with at least three biological replicates showing similar results. SP, signal peptide; WT, wild type.

### Identification of pathogenic effector genes in R0301

Considering R0301 is a highly pathogenic endemic isolate of *Rc* in China since 2010 [7, 34, 35], we further analyzed effector genes in R0301. Previous studies demonstrated that genes upregulated during fungal infection generally have roles in pathogenicity [13]. To detect the key effector genes, we sequenced the transcriptome of in vitro R0301 mycelia and infected wheat seedlings at 3‐, 5‐, and 7‐days post inoculation (dpi) using the genome information of R0301 from the study as a reference. We detected a total of 13,675 expressed genes, among which 4435 were identified as differentially expressed genes (DEGs) from pairwise comparisons of 0 dpi versus 3 dpi, 0 dpi versus 5 dpi, and 0 dpi versus 7 dpi (Figure 3C). GO class analysis largely assigned these DEGs in catalytic activity, binding, membrane, membrane part, and metabolic process (Figure 3D). Of these DEGs, 2402 were significantly upregulated and were presumed potential *Rc* pathogenicity genes (Figure 3C and Table S8). GO enrichment analysis showed that these upregulated genes were mainly enriched in carbohydrate metabolic processes, integral component of membrane, and oxidoreductase activity (Figure S4A–C). The Kyoto encyclopedia of genes and genomes (KEGG) analysis showed that these upregulated genes were mainly involved in a variety of metabolic pathways, such as starch and sucrose metabolism, tryptophan metabolism, and pentose and glucuronate interconversions (Figure S4D). Combined with the predicted 98 effector genes from isolate R0301, 43 were significantly upregulated after infection (Figure 3A,B), and some of them with conserved domains included DPBB (double‐psi beta‐barrel), RlpA (rare lipoprotein A) EXP (expansins)_N (N‐terminal)‐like domain, glycoside hydrolase family, LPMO (lytic polysaccharide mono oxygenase) auxiliary activity family, pectate lyase, and SGNH (Ser‐Gly‐Asn‐His) hydrolase subfamily (Table S9). These results suggested that any of the 43 upregulated effector genes could be involved in *Rc* pathogenicity, and they were therefore nominated as potential effector candidates in R0301.

### Genome‐wide association study (GWAS) of *Rc* pathogenicity

To further uncover the candidate genes associated with *Rc* pathogenicity, the 207 *Rc* isolates with genome resequencing data were selected for GWAS. The sharp eyespot disease index (DI) of the 207 *Rc* isolates on four host genotypes showed continuous variation from 13.8 to 81.5 (Table S1 and Figure S5A−G). GWAS results for pathogenicity using the SNPs identified in the *Rc* genomic variant atlas revealed SNPs distributed on all chromosomes except chromosome 4 (Chr. 4); 16 significant SNPs were identified by at least two repeated experiments (Table S10, Figures 3E and S5H−S). The decay distance of linkage disequilibrium (LD) for 207 *Rc* isolates was estimated at 0.48 kb (Figure S5T). Based on our LD decay results and previous studies [36, 37, 38], we used 200‐Kb intervals covering significant SNP as the possible range of candidate genes associated with *Rc* pathogenicity. Consequently, 767 genes were included in the 200‐Kb intervals of 16 significant SNPs based on the genome of R0301. Considering the 43 upregulated effector genes in R0301, only EVM0004929 (designated as *RcPL3*) was identified by GWAS (Table S11). The sequencing results in *Rc* isolates revealed that the *RcPL3* coding region contained 12 variants forming nine haplotypes. Comparison of pathogenicity showed that DI of isolates with *RcPL3_HapB* was relatively lower than that of *RcPL3_HapA* (Table S12). qRT‐PCR showed that *RcPL3* was significantly induced in wheat plants at 5 dpi with R0301 (Figure 3F). These results suggested that *RcPL3* was associated with *Rc* pathogenicity.

### RcPL3 plays a central effector role in *Rc* pathogenicity

Deduced amino acid analysis showed that RcPL3 was a secreted protein, enriched in glycine (10.89%) and threonine (10.12%), with a signal peptide (SP) in its N‐terminus (Figure 3G). This was supported by the Alphafold2 analysis (Figure 3H). To verify the SP function of RcPL3, we constructed a SUC2‐RcPL3SP vector and transformed it into the yeast strain YTK12 without a secreted invertase. The SUC2‐RcPL3SP transformants successfully grew on SD‐Trp‐glucose agar and YPRAA plates (Figure 3I). Secreted invertase activity by the transformant containing RcPL3SP was detected by reduction of 2,3,5‐triphenyltetrazolium chloride (TTC) (Figure 3I). These results confirmed that the RcPL3 signal peptide is functional and capable of directing invertase secretion in yeast.

Suppression of hypersensitive response (HR) is usually regarded as an important criterion for decreasing plant immunity. To verify whether RcPL3 suppresses BAX‐induced programmed cell death (PCD) related to HR in plant cells, we cloned RcPL3 fragments without a SP from isolate R0301, and generated RcPL3^ΔSP^ construct. In *N. benthamiana* leaves infiltrated with RcPL3^ΔSP^ alone, no visual cell death was apparent, whereas cell death was observed in leaves infiltrated with BAX. However, on leaves infiltrated with RcPL3^ΔSP^ 24 h before infiltration with BAX, cell death was not observed (Figure 3J). The statistical analysis results showed that lesion areas of tobacco leaves with transient *RcPL3* overexpression were significantly smaller than controls (Figure S5U). These results indicated that RcPL3 significantly suppresses BAX‐induced PCD in *N. benthamiana*.

To verify the function of *RcPL3* during *Rc* infection of wheat plants, we performed virus‐induced gene silencing (VIGS) experiment mediated by the barley strip mosaic virus (BSMV) in the SE‐susceptible wheat cultivar YM49 (Figures 3K and S6A). qRT‐PCR detected significantly reduced expression of *RcPL3* and *RcOSP1* (positive controls) [39] in silenced plants (Figures 3L and S6B), implying that the transient BSMV‐mediated VIGS successfully silenced target genes in *Rc* during its infection of wheat. After inoculation by *Rc* (R0301), the *RcPL3*‐silenced plants and positive controls exhibited significant reductions in sharp eyespot DI (Figures 3M and S6C) and the malondialdehyde (MDA) content compared with the negative controls (BSMV:00) (Figure S6D), implied that silencing of *RcPL3* gene significantly decreased *Rc* pathogenicity. Moreover, the superoxide dismutase (SOD), catalase (CAT), and peroxidase (POD) contents had sharply increased in the *RcPL3*‐silenced plants (Figure S6E–G). These results suggest that *RcPL3* may play an important role in the infection of wheat plants.

To further confirm the pathogenicity of *RcPL3*, we constructed an overexpression (OE) vector, *RcPL3*‐LGY‐OE3, and transformed it into the wheat cultivar Fielder. After positive detection by specific primers, three T_0_ *RcPL3*‐OE plants with relatively high protein levels detected by Western blotting were self‐crossed into the T_2_ lines. After inoculation by *Rc*, three *RcPL3*‐OE lines exhibited significantly increased sharp eyespot DI (Figure S7A−C), mycelial coverage and cell death (Figure S7D), hyphal growth (Figure S7E), as well as increased H_2_O_2_ contents (Figure S7F) compared with controls. These results imply that *RcPL3* is a key pathogenicity gene during *Rc* infection in wheat plants.

### RcPL3 causes the accumulation of TaHDA9 protein in the cytoplasm of wheat

To dissect the pathogenicity mechanism of *RcPL3*, we used RcPL3 as bait to screen for interacting host proteins in the wheat cDNA library by yeast two‐hybrid (Y2H) assays. Of the screened 12 different candidates, a histone deacetylase (TaHDA9, TraesCS2B03G0808200) was selected due to its high frequent presence in the screened clients and high expression in the basal stem of wheat (Figure S8A) as well as its role of regulating plant development and immunity as a histone deacetylase in plants [40, 41, 42, 43]. Interaction between RcPL3 and TaHDA9 in yeast cells by Y2H assays (Figure 4A) and in *N. benthamiana* cells by a split firefly luciferase complementation imaging (LCI) assays (Figure 4B) was subsequently confirmed. Pull‐down assays showed that GST‐TaHDA9 pulled down His‐RcPL3 after GST‐tag protein purification (Figure 4C). Bimolecular fluorescence complementation (BiFC) analysis in wheat mesophyll protoplasts demonstrated that RcPL3 and TaHDA9 interacted in the cytoplasm (Figure 4D). These results indicated that RcPL3 interacted with TaHDA9 in vivo and in vitro. Additionally, TaHDA9 interacted with both RcPL3_HapA and RcPL3_HapB by Y2H (Figure S8B), implying that *RcPL3* is a relatively conserved effector.

**FIGURE 4:**
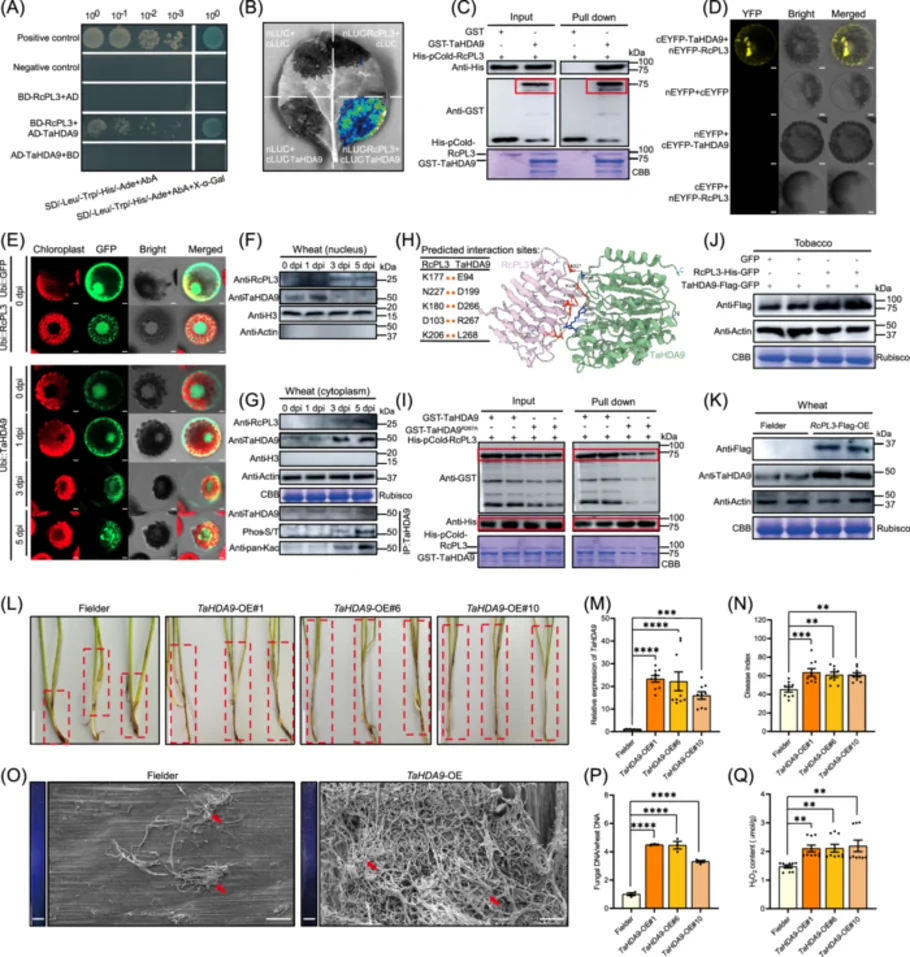
RcPL3 recruits TaHDA9 through physical interaction to promote host susceptibility to *Rc*. (A−D) Interaction of RcPL3 and TaHDA9 verified by yeast two‐hybrid assay (Y2H) (A), firefly luciferase complementation imaging (LCI) in *N. benthamiana* (B), pull‐down (C), and bimolecular fluorescence complementation (BiFC) (D), respectively. The red box in (C) indicates TaHDA9. (E) Subcellular localization of the Ubi::GFP‐RcPL3 and Ubi::GFP‐TaHDA9 fusion protein in wheat protoplast at 0 dpi, and subcellular localization of the Ubi::GFP‐TaHDA9 fusion protein in wheat protoplast at 0, 1, 3, and 5 dpi with R0301. GFP fusion protein acts as the controls. Green signal indicates fluorescence from GFP fusion protein, while red signal shows intrinsic chloroplast auto‑fluorescence of wheat protoplasts. Bright, bright‑field channel; Merged, composite overlay of individual channels. Immunoblots detect protein levels of RcPL3 and TaHDA9 in wheat cell nucleus (F) and cytoplasm (G), respectively, at 0, 1, 3, and 5 dpi with R0301, respectively. (H) The residues (E94, D199, D266, R267, and L268) at five positions in TaHDA9 are the important sites interacting RcPL3 by Alphafold2 analysis. (I) The pull‐down assay indicates that R267A of TaHDA9 significantly weakened the interaction of RcPL3 and TaHDA9. The red box indicated TaHDA9, TaHDA9^R267A^, and RcPL3. (J and K) RcPL3 increases the accumulation of TaHDA9 protein. Immunoblots detecting transient expression of 35S::TaHDA9‐Flag‐GFP with (+) or without (−) 35S::RcPL3‐His‐GFP in *N. benthamiana* leaves cells (J); Immunoblots detect the protein abundance of TaHDA9 in WT and *RcPL3‐Flag*‐OE wheat plants (K). *TaHDA9*‐OE lines significantly decrease SE resistance. Comparison of disease response (L), relative expression levels of *TaHDA9* (M), SE disease index (N) (12 plants per replicate, *n* = 9), trypan blue staining of necrotic cell death and mycelial coverage (O), *Rc* biomass (P), and H_2_O_2_ content (Q) in WT and *TaHDA9*‐OE lines. Scale bars, 5 μm in (D, E), 1 cm in (L), and 100 μm in (O). Red arrows in (O) showed the *Rc* hypha. Data in (M and N) and (P and Q) are presented as the mean values ± SE with individual data points (**p < 0.01 and ****p < 0.0001; one‐way ANOVA with Dunnett's multiple comparisons test). In (A−G), (I−L), and (O), the experiments were conducted with at least three biological replicates showing similar results. GFP, green fluorescent protein.

To localize RcPL3 and TaHDA9 in cells, we performed subcellular localization in wheat protoplasts using transient expression vectors Ubi::GFP‐RcPL3 or Ubi::GFP‐TaHDA9. Results showed that GFP signals of RcPL3 and TaHDA9 were both observed in almost the whole cell (Figure 4E). Subsequently, we performed subcellular localization in wheat protoplasts at 0, 1, 3, and 5 dpi, respectively. The results showed that the signals of TaHDA9 in the nucleus gradually weakened, while its signals in the cytoplasm gradually increased with increasing infection time (Figure 4E). We next synthesized TaHDA9‐ and RcPL3‐specific antibodies. Phylogenetic analysis indicated that TaHDA9 exhibited lower sequence similarity with other HDAC members, except for its homologous genes, demonstrating the specificity of the TaHDA9 antibody (Figure S8C,D). We extracted proteins from the nucleus and cytoplasm of wheat cell during *Rc* infection, respectively. Immunoblotting results using anti‐RcPL3 and anti‐TaHDA9 specific antibodies showed that the abundance of RcPL3 protein was significantly improved in both nucleus and cytoplasm during infection (Figure 4F,G). However, TaHDA9 protein level was significantly decreased in the nucleus and could not even be detected at 3 dpi, but was increased significantly in the cytoplasm during *Rc* infection (Figure 4F,G). As the level of RcPL3 protein increased, the phosphorylation and acetylation (Kac) levels of TaHDA9 were significantly promoted (Figure 4G). These results suggest that the TaHDA9 protein accumulates gradually in the cytoplasm during *Rc* infection possibly through PTM.

Alphafold2 analysis suggests that five residues (E94, D199, D266, R267, and L268) in TaHDA9 are the important interaction sites of RcPL3 and TaHDA9 protein complex (Figure 4H). Next, we created six mimic mutants (E94A, D199A, D266A, R267A, L268A, and D266A/R267A/L268A) of TaHDA9 (Figure S9A). Pull‐down assays showed that TaHDA9^E94A^, TaHDA9^D199A^, TaHDA9^R267A^, TaHDA9^L268A^, and TaHDA9^D266A/R267A/L268A^ substantially weakened the interaction with RcPL3, especially TaHDA9^R267A^ and TaHDA9^D266A/R267A/L268A^ (Figures 4I and S9B−I, Table S13), suggesting that the R267 is possibly a key point of interaction between TaHDA9 and RcPL3.

To determine whether RcPL3 affects the protein accumulation of TaHDA9, we transiently co‐expressed vector 35S::TaHDA9‐Flag‐GFP with 35S::GFP or 35S::RcPL3‐His‐GFP in *N. benthamiana* leaves. Immunoblots using anti‐Flag revealed that the presence of RcPL3 significantly increased the abundance of TaHDA9 (Figure 4J and Table S13). Next, we extracted total proteins from *RcPL3*‐OE wheat plants and immunoblotting results confirmed increased accumulation of TaHDA9 in *RcPL3*‐OE plants (Figure 4K and Table S13). These results suggested that RcPL3 promoted *Rc* infection possibly through recruiting TaHDA9.

### *TaHDA9* negatively regulates SE resistance in wheat

To verify a role of *TaHDA9* in SE resistance, we silenced *TaHDA9* in the susceptible wheat cultivar YM49 by BSMV‐mediated VIGS (Figure S10A). qRT‐PCR results indicated that the expression level of *TaHDA9* was significantly decreased in *TaHDA9*‐silenced plants compared with the controls (Figure S10B). *TaHDA9*‐silenced plants had significantly lower DI than controls after infection by *Rc* (Figure S10C). These results suggest that *TaHDA9* negatively regulates SE resistance in wheat.

To further illustrate the influence of silencing *TaHDA9* on SE resistance, we next screened two mutants (K733, a splice acceptor variant; K2217, a premature stop codon) of *TaHDA9* gene in the EMS‐mutagenized tetraploid wheat Kronos libraries (Figure S10D). To exclude other mutation sites, we backcrossed them into BC_2_ lines with wild type (WT) for two times. When infected with *Rc*, both mutants had significantly decreased DI and significantly reduced H_2_O_2_ concentrations compared with the controls (Figure S10E−H). These results showed that the mutation of *TaHDA9* led to increased SE resistance in tetraploid wheat.

A wheat overexpression vector, *TaHDA9*‐LGY‐OE3, containing the full‐length cDNA of *TaHDA9* was transformed into the moderately susceptible cultivar Fielder. After positive detection, we quantified expression levels of *TaHDA9* in positively transgenic lines by qRT‐PCR, and selected three highly expressed plants to self‐cross into T_2_ generation, and further inoculated with *Rc* (Figure 4L,M). The results showed that the *TaHDA9*‐OE lines exhibited significantly higher DI (Figure 4N), more mycelial coverage (Figure 4O) and Rc biomass (Figure 4P), and significantly increased cell death (Figure 4O) and H_2_O_2_ contents (Figure 4Q) compared with controls. These results indicated that *TaHDA9* negatively modulated SE resistance in wheat, possibly by mediating reactive oxygen species (ROS) accumulation. Moreover, we used the CRISPR/Cas9 gene‐editing technique to knock out *TaHDA9* gene in Fielder and confirmed mutations by Sanger sequencing (Figure S10I,J). After *Rc* infection, *TaHDA9*‐edited wheat lines exhibited significantly reduced mycelial coverage (Figure S10K), lower DI (Figure S10L), decreased H_2_O_2_ contents (Figure S10M), and less *Rc* biomass (Figure S10N) compared with the controls. These results suggest that *TaHDA9* negatively modulates wheat SE resistance.

Sequencing results in 480 wheat accessions revealed that *TaHDA9* exon possessed four variants mainly forming three haplotypes *TaHDA9*_*HapA*, *TaHDA9*_*HapB*, and *TaHDA9*_*HapC*. Comparison of phenotypes indicated that accessions with *TaHDA9*_*HapB* exhibited significantly stronger SE resistance than those with *TaHDA9*_*HapA* (Table S12). These results suggest that *TaHDA9*_*HapB* could potentially be used to improve SE resistance in wheat breeding program.

To check whether R267A mutation abolishes the ability of RcPL3, we constructed the wheat vector Ubi::LGY‐OE3‐TaHDA9^R267A^ and transferred it into wheat for overexpression. After infection by *Rc*, the *TaHDA9* ^R267A^‐OE plants exhibited a significantly lower DI (Figure S10O−Q), decreased *Rc* biomass (Figure S10R), and H_2_O_2_ contents (Figure S10S) compared with the controls. Thus, these results suggest that the *TaHDA9* ^R267A^ leads to improved disease resistance in wheat possibly due to the R267A mutation escaping the recruitment by RcPL3.

### TaHDA9 reduces the accumulation of TaRHC1A protein possibly by deacetylation

#### TaHDA9 interacts with TaRHC1A

To dissect the molecular mechanism regulating SE resistance, we used TaHDA9 as bait to screen for interacting proteins in the wheat cDNA library by Y2H analysis. Of the 65 screened different clients, TaRHC1A (TraesCS3D03G0732400) was selected due to its high frequent presence in the screened clients and high expression in the basal stem of wheat (Figure S8A), as well as the characterization of RING proteins that were previously reported to play integral roles in plant immunity [44, 45, 46]. Interaction of TaHDA9 and TaRHC1A was confirmed in yeast by Y2H assays (Figure 5A), in *N. benthamiana* leaves by LCI (Figure 5B), and in wheat plants by co‐immunoprecipitation (Co‐IP) (Figure 5C). In vitro pull‐down assays indicated that GST‐TaHDA9 pulled down TaRHC1A after GST‐tag protein purification (Figure 5D). Subcellular localization in wheat protoplasts showed that Ubi::GFP‐TaRHC1A was localized in the cytoplasm (Figure 5E). BiFC assay in wheat mesophyll protoplasts showed that TaHDA9 interacted with TaRHC1A in the cytoplasm (Figure 5F). These results indicated that TaHDA9 interacted with TaRHC1A in vivo and in vitro.

**FIGURE 5:**
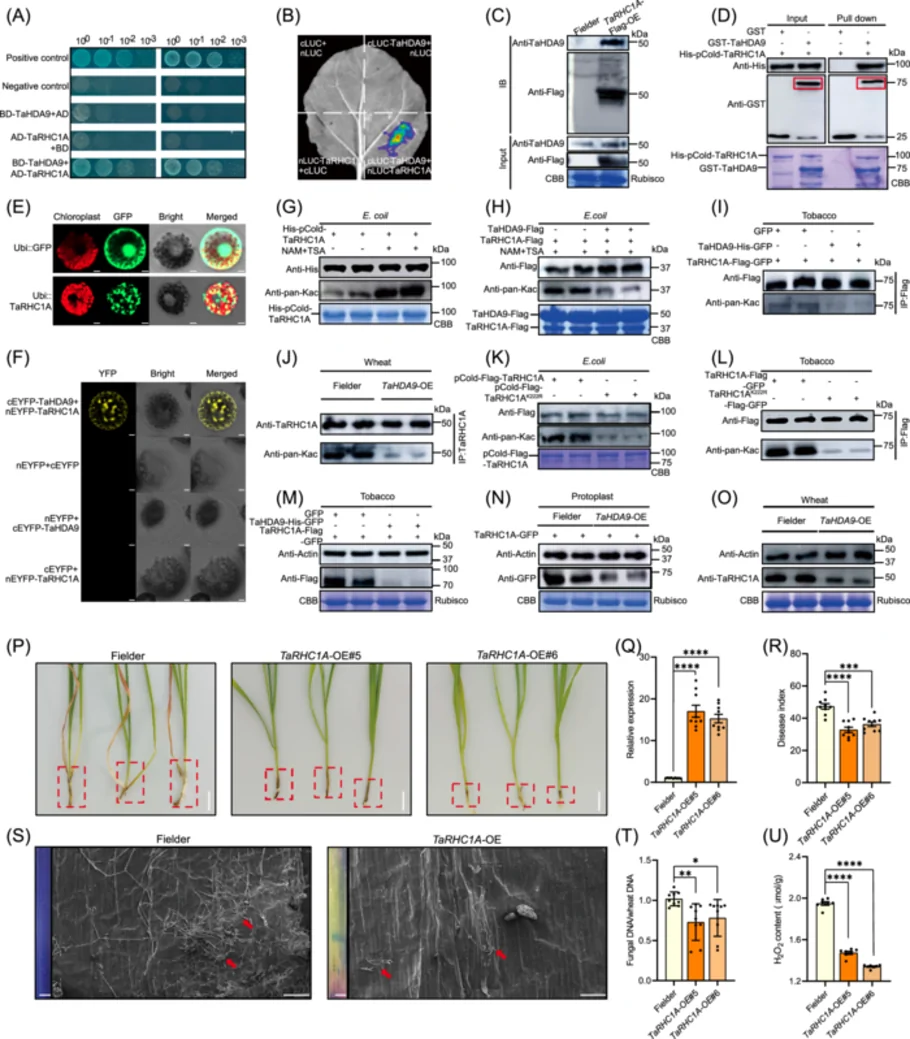
TaHDA9 inhibits the level of TaRHC1A protein possibly by deacetylation, and *TaRHC1A* positively regulates sharp eyespot response in wheat. (A−D) Interaction of TaHDA9 and TaRHC1A verified by Y2H (A), firefly LCI in *N. benthamiana* (B), co‐immunoprecipitation (C), and pull‐down (D). The red box in (D) indicates TaHDA9. IB, Immunoblotting. (E) Subcellular localization of Ubi::GFP‐TaRHC1A fusion protein in wheat protoplast. GFP fusion protein acts as the controls. (F) Interaction of TaHDA9 and TaRHC1A verified by BiFC. (G) Recombinant His‐pCold‐TaRHC1A protein purified from *E. coli* is acetylated, and an *E. coli* strain containing the His‐pCold‐TaRHC1A construct is cultured in LB medium with or without richostatin A (TSA) and *β*‐nicotinamide (NAM). The acetylation status of TaRHC1A is determined with a pan‐Kac antibody (anti‐acetylated lysine antibody). (H) TaRHC1A is deacetylated by TaHDA9 in vitro. Lysine acetylation (Kac) status of TaRHC1A together with (+) or without (−) TaHDA9 in *E. coli* cells determined by immunoblots in vivo. (I and J) TaHDA9 reduces the Kac level of TaRHC1A in vivo. Immunoblot detection of Kac levels of TaRHC1A with (+) or without (−) TaHDA9 in *N. benthamiana* cells (I). Immunoblots detect Kac levels of TaRHC1A with anti‐TaRHC1A specific antibody in *TaHDA9*‐OE lines and controls (J). (K, L) The K222R change significantly decreased the Kac level of TaRHC1A. Kac levels of TaRHC1A and TaRHC1A^K222R^ in *E. coli* cells determined by immunoblots (K). Immunoblot detection of Kac levels of TaRHC1A or TaRHC1A^K222R^ in *N. benthamiana* leaves cells (L). (M−O) TaHDA9 reduces the accumulation of TaRHC1A protein. Immunoblots detect transient expression of 35S::TaRHC1A‐Flag‐GFP with (+) or without (−) 35S::TaHDA9‐His‐GFP in *N. benthamiana* leaves cells (M); Immunoblots detect transient expression of Ubi:TaRHC1A‐GFP in Wild‐type (Fielder) and *TaHDA9*‐OE wheat leaf protoplasts (N). Immunoblots detect that the protein abundance of endogenous TaRHC1A is significantly decreased in *TaHDA9‐OE* plants (O). (P−U) *TaRHC1A*‐OE lines had significantly increased wheat SE resistance. Relative expression levels of the *TaRHC1A* gene in WT (Fielder) and *TaRHC1A‐OE* lines (*TaRHC1A*‐OE*#5* and *TaRHC1A*‐OE*#6*) (Q). Comparison of disease response (P), SE disease index (R) (12 plants per replicate, *n* = 9), trypan blue staining of necrotic cell death and mycelial coverage (S), *Rc* biomass (T), and H_2_O_2_ content (U) in WT and *TaRHC1A*‐OE lines. Scale bars, 5 μm in (E, F), 1 cm in (P), and 100 μm in (S). Red arrow in (S) showed the *Rc* hypha. Data in (Q and R) and (T and U) are presented as the mean values ± SE with individual data points (*p < 0.05, **p < 0.01, ***p < 0.001 and ****p < 0.0001; one‐way ANOVA with Dunnett's multiple comparisons test). In (A−P) and (S), the experiments were conducted with at least three biological replicates showing similar results.

#### TaHDA9 deacetylates TaRHC1A

As TaHDA9 is a histone deacetylase, we assumed that TaRHC1A could be deacetylated by TaHDA9. We induced prokaryotic expression of His‐tagged TaRHC1A in *E. coli* and measured TaRHC1A Kac levels using an anti‐acetylated lysine antibody (anti‐Kac antibody). Immunoblotting results showed that the Kac level of TaRHC1A was significantly enhanced after treatment with deacetylation inhibitors trichostatin A (TSA) and *β*‐nicotinamide (NAM) (Figure 5G), implying TaRHC1A could be acetylated when ectopically expressed in *E. coli* cells. In vitro lysine deacetylation assays of purified TaRHC1A and TaHDA9 proteins in *E. coli* showed that the Kac level of TaRHC1A was decreased with addition of TaHDA9 protein in *E. coli* cultures treated with TSA and NAM (Figure 5H). To determine whether TaHDA9 deacetylates TaRHC1A in vivo, we transiently co‐expressed fusion vectors 35S::TaRHC1A‐Flag‐GFP with 35S::GFP or 35S::TaHDA9‐His‐GFP in tobacco leaf cells, respectively. Immunoblotting results indicated that the addition of TaHDA9 significantly reduced the Kac level of TaRHC1A (Figure 5I). Thus, TaHDA9 possibly deacetylated TaRHC1A in *N. benthamiana*. Subsequently, we extracted proteins from *TaHDA9*‐OE lines and controls, respectively. Immunoblotting results with anti‐TaRHC1A specific antibody indicated that TaRHC1A exhibited decreased Kac levels in *TaHDA9*‐OE lines compared with the controls (Figure 5J). These results demonstrated that TaRHC1A could be deacetylated by TaHDA9 in vitro and in vivo.

We subsequently performed mass spectroscopy analysis of *E. coli*‐produced TaRHC1A to identify the Kac site in TaRHC1A. Results indicated that one lysine residue (K222) in TaRHC1A was modified by Kac in the absence of TaHDA9 (Figure S11A). We mutated K222 and K11 (negative control) into arginine (R222, R11) to generate the deacetylated mutants TaRHC1A^K222R^ and TaRHC1A^K11R^ (Figure S11B,C), respectively. In vitro differential Kac assays in *E. coli* showed that K222R mutation significantly decreased the Kac level of TaRHC1A (Figure 5K and Table S13), but K11R mutation did not show significant difference (Figure S11D and Table S13). We then transiently overexpressed *TaRHC1A* and *TaRHC1A* ^*K222R*^ in *N. benthamiana*, respectively, and immunoblotting results showed that the Kac level of TaRHC1A^K222R^ was substantially reduced (Figure 5L and Table S13). These results suggest that K222 is a key site in TaRHC1A deacetylated by TaHDA9.

#### TaHDA9 reduces the accumulation of TaRHC1A protein by deacetylation

To determine if TaHDA9 affects the level of TaRHC1A protein, we transiently co‐expressed vectors 35S::TaRHC1A‐Flag‐GFP and 35S::GFP or 35S::TaHDA9‐His‐GFP in *N. benthamiana* leaf cells, respectively. Immunoblotting results using anti‐Flag antibody revealed that the addition of TaHDA9 significantly reduced the abundance of TaRHC1A (Figure 5M). Immunoblotting of transiently overexpressed Ubi::TaRHC1A‐GFP in protoplasts from wheat leaves using anti‐GFP antibody revealed that TaRHC1A protein accumulation was further decreased in *TaHDA9*‐OE plants (Figure 5N). Next, we extracted total proteins from *TaHDA9*‐OE wheat plants and control, and immunoblotting results confirmed decreased protein level of TaRHC1A in *TaHDA9*‐OE plants (Figure 5O).

To further test whether the deacetylation at K222 affects TaRHC1A protein accumulation by TaHDA9, we first extracted total proteins from *TaHDA9*‐OE lines and controls, and then purified TaRHC1A and TaRHC1A^K222R^ proteins from *E. coli*. We then mixed the wheat total proteins together with TaRHC1A or TaRHC1A^K222R^ to determine their levels at different stages by the cell‐free system (Figure S11E−H). Immunoblotting results using an anti‐His antibody showed that TaRHC1A protein accumulation was sharply decreased in *TaHDA9*‐OE plants compared with the control after 10 min, indicating that TaHDA9 could decrease TaRHC1A protein accumulation (Figure S11E,F). However, the TaRHC1A^K222R^ protein level decreased more sharply than that of TaRHC1A in Fielder (Figure S11E,G), indicating the decreased protein accumulation through the deacetylation of K222 in TaRHC1A. The TaRHC1A^K222R^ protein level decreased sharply in *TaHDA9*‐OE and WT plants (Figure S11G,H), suggesting that TaRHC1A^K222R^ is not deacetylated by TaHDA9. These results indicated that TaHDA9 decreased the accumulation of TaRHC1A through deacetylating K222.

Meanwhile, we transiently overexpressed Ubi::TaRHC1A‐GFP and Ubi::TaRHC1A^K222R^‐GFP in the protoplasts of *TaRHC1A*‐OE lines and WT (Fielder) plants, respectively. Immunoblotting results showed that the TaRHC1A^K222R^ exhibited significantly lower protein levels in both Fielder and *TaRHC1A*‐OE plants (Figure S11I). Additionally, immunoblotting results using an anti‐His antibody revealed that adding MG132 significantly increased the abundance of TaRHC1A^K222R^ protein in Fielder (Figure S11I). We then transiently expressed vector 35S::TaRHC1A^K222R^‐Flag‐GFP in *N. benthamiana*, and extracted total proteins to determine TaRHC1A^K222R^ level at different stages by the cell‐free system (Figure S11J). Immunoblotting results with anti‐Flag antibody indicated that adding MG132 significantly increased the abundance of TaRHC1A^K222R^ protein. These results suggest the degradation of TaRHC1A^K222R^ through the 26S proteasome pathway.

### *TaRHC1A* promotes the degradation of TaRBOH3 by ubiquitination via 26S proteasome to reduce ROS content

To verify its role in regulating SE resistance, we silenced *TaRHC1A* in the SE‐resistant cultivar PY50 using BSMV‐mediated VIGS (Figure S11K). qRT‐PCR analysis showed that the *TaRHC1A* expression level was significantly decreased in BSMV_TaRHC1A_ plants (Figure S11L), and the *TaRHC1A*‐silenced plants exhibited significantly higher DI than controls after infection by *Rc* (Figure S11M). We then screened a mutant line (K3490, with a premature stop codon; Figure S11N,O) of the *TaRHC1A* gene in the EMS‐mutagenized tetraploid wheat Kronos libraries. To exclude the possibility of mutations at other mutation sites, we backcrossed K3490 with the WT twice to obtain the BC_2_ mutant (*rhc1a*). After inoculation by *Rc*, the *rhc1a* mutant exhibited a higher DI (Figure S11P) and significantly higher H_2_O_2_ concentrations (Figure S11Q) compared to the WT.

To further confirm the role of *TaRHC1A* in SE resistance, we constructed a wheat overexpression vector pUbi::LGY‐OE3 containing the full‐length cDNA of the *TaRHC1A*, and transformed it into Fielder (Figure 5P). After positive detection, we quantified expression levels of *TaRHC1A* in positively transgenic lines by qRT‐PCR, and selected two highly expressed plants (Figure 5Q) to self‐cross into T_2_ generation, and further inoculated with *Rc*. When infected with *Rc*, these lines showed significantly lower DI (Figure 5R), as well as reduced mycelial coverage and cell death (Figure 5S), *Rc* biomass (Figure 5T), and H_2_O_2_ contents (Figure 5U) compared to the controls. These results suggest that *TaRHC1A* overexpression increase SE resistance due to ROS scavenging.

To investigate the influence of *TaRHC1A* on ROS, we separately infiltrated *Agrobacterium* harboring a 35S::TaRHC1A‐Flag‐GFP and an empty vector 35S::GFP into *N. benthamiana* leaves with the co‐infiltration of *P. syringae* strain DC3000, respectively. ROS content in leaves of the *TaRHC1A*‐OE plants was substantially reduced compared with the control (DC3000 + GFP) (Figure 6A,B), indicating that *TaRHC1A* was able to scavenge ROS in *N. benthamiana*. However, with transient overexpression of *TaHDA9* in *TaRHC1A*‐OE plants, ROS content was restored compared with control (DC3000+TaRHC1A) (Figure 6B,C), indicating that TaHDA9 inhibited TaRHC1A scavenging ROS *in planta*. Furthermore, when *TaHDA9*, *TaRHC1A*, and *RcPL3* were co‐expressed, ROS accumulation was sharply increased compared with the controls (DC3000 + TaRHC1A + TaHDA9 or DC3000 + TaRHC1A) (Figure 6C,D). These results implied that the increased accumulation of TaHDA9 by adding RcPL3 substantially inhibited TaRHC1A to scavenge ROS, finally leading ROS burst.

**FIGURE 6:**
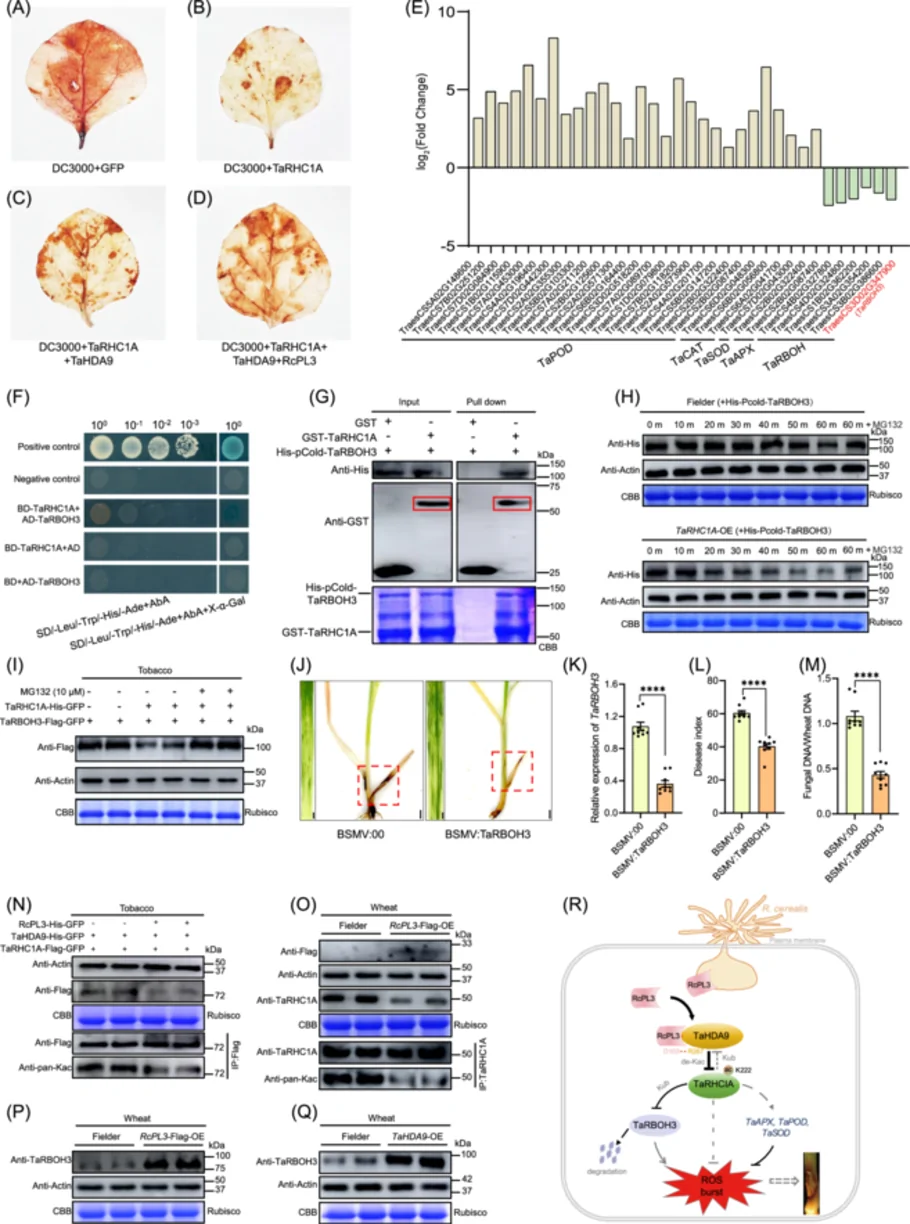
RcPL3 as a key effector recruits TaHDA9 that suppresses TaRHC1A by deacetylation and thus inhibits the degradation of TaRBOH3 by TaRHC1A. (A−D) 3,3’‐diamino benzidine hydrochloride (DAB) staining shows that TaRHC1A can scavenge reactive oxygen species (ROS) accumulation in *N. benthamiana*, but the capacity of eliminating ROS is inhibited by TaHDA9 and RcPL3. DC3000 + GFP, positive control; TaRHC1A, 35S::TaRHC1A‐Flag‐GFP; TaHDA9, 35S::TaHDA9‐His‐GFP; RcPL3, 35S::RcPL3‐His‐GFP. (E) Transcriptome sequencing identifies 6 down‐regulated and 28 up‐regulated differentially expressed genes (DEGs) involved in ROS. Interaction of TaRHC1A and TaRBOH3 verified by Y2H (F) and pull‐down (G). The red box in (G) indicates TaRHC1A. (H) The protein level of TaRBOH3 is decreased in *TaRHC1A*‐OE plants possibly through the 26S proteasome pathway using the cell‐free system. (I) TaRHC1A reduces the TaRBOH3 protein amount possibly through the 26S proteasome pathway in *N. benthamiana* leaf cells. VIGS verifies the function of *TaRBOH3*. Phenotypes (J), relative expression levels (K), disease index (L) (12 plants per replicate, *n* = 9), *Rc* biomass (M) of BSMV:00, and BSMV:TaRBOH3 plants. (N) RcPL3 decreases the acetylation level and abundance of TaRHC1A in the presence of TaHDA9 in *N. benthamiana*. (O) Immunoblots detect that the acetylation level and abundance of endogenous TaRHC1A are decreased in *RcPL3*‐OE plants. Immunoblots detect that the protein abundance of endogenous TaRBOH3 is significantly increased in *RcPL3*‐OE plants (P) and *TaHDA9*‐OE plants (Q). (R) Proposed model of the RcPL3‐mediated *Rc* infection in wheat. Scale bars, 2 mm for the leaf and 1 cm for the whole plant in (J). Data in (K−M) are presented as the mean ± SE with individual data point (****p < 0.0001; Unpaired two‐sided *t*‐test with at least three biologically independent replicates). In (A−D), (F−J), and (N−Q), the experiments were conducted with at least three biological replicates showing similar results. de‐Kac, lysine deacetylation; Kub, lysine ubiquitination.

Transcriptome sequencing of *TaRHC1A*‐OE wheat plants indicated that 6081 of identified 133,382 genes were differentially expressed compared with the WT (Figure S12A). Further analysis showed that 37 of 868 antioxidant enzyme genes were differentially expressed, and 28 of them were significantly up‐regulated, including 22 peroxidase (*TaPOD*), 3 catalase (*TaCAT*), 2 ascorbate peroxidase (*TaAPX*), and 1 superoxide dismutase (*TaSOD*) genes (Figure 6E). Concurrently, six DEGs (respiratory burst oxidase genes, *TaRBOH*) responsible for ROS synthesis were significantly down‐regulated (Figure 6E). We next detected ROS‐related enzyme activity in *TaRHC1A*‐OE and control plants. The results showed that the APX and POD activities were increased sharply in the *TaRHC1A*‐OE plants (Figure S12B,C). These results suggested that *TaRHC1A*‐OE plants decreased ROS, possibly by promoting the accumulation of antioxidant enzymes‐mediating ROS removal and inhibiting RBOH‐mediated ROS synthesis.

To verify this hypothesis, we used TaRHC1A as bait to screen for interacting proteins in the wheat cDNA library by Y2H analysis. A client TaRBOH3 (TraesCS3D03G0772900) as a member of RBOH family was selected due to its high frequent presence in the screened clients and high expression in the basal stem of wheat (Figure S8A) as well as the *RBOH*‐mediated ROS signaling in plant immunity [47]. We next confirmed the interaction of TaRHC1A and TaRBOH3 proteins in the yeast cells by Y2H (Figure 6F) and in vitro pull‐down (Figure 6G). Alphafold2 revealed TaRHC1A possibly interacting with TaRBOH3 (Figure S13A). These results indicated that TaRHC1A strongly interacted with TaRBOH3 both in vivo and in vitro. To test whether TaRHC1A affects TaRBOH3 protein amount, we first extracted total proteins from *TaRHC1A*‐OE lines and controls and purified the His‐pCold‐TaRBOH3 protein from *E. coli*. Then, we mixed these proteins together to determine the level of TaRBOH3 protein in a cell‐free system at different stages. Immunoblotting results with anti‐His antibody indicated that the TaRBOH3 protein amount was sharply decreased in *TaRHC1A*‐OE plants compared with WT after 20 min, however, after adding MG132, the decrease of TaRBOH3 was significantly inhibited (Figure 6H). Subsequently, we extracted proteins from *TaRHC1A*‐OE lines and as well as controls. Immunoblotting results with anti‐TaRBOH3 specific antibody indicated that TaRBOH3 exhibited decreased protein abundance in *TaRHC1A*‐OE lines compared with the controls (Figure S13B). Therefore, TaRBOH3 is possibly degraded through polyubiquitination by the E3 ligase TaRHC1A. To verify this hypothesis, we transiently co‐expressed TaRBOH3 with or without TaRHC1A in *N. benthamiana* cells. Western blot using an anti‐Flag antibody showed that the addition of TaRHC1A significantly reduced the protein level of TaRBOH3; however, after adding MG132, the decrease of TaRBOH3 was significantly inhibited (Figure 6I). These results confirmed that TaRHC1A reduced the protein amount of TaRBOH3 in *N. benthamiana* and wheat.

Subsequently, we silenced *TaRBOH3* in YM49 by BSMV‐mediated VIGS (Figure 6J). qRT‐PCR detected significantly decreased expression of *TaRBOH3* in BSMV_*TaRBOH3*_ plants compared with the controls (Figure 6K). After infection by *Rc*, *TaRBOH3*‐silenced plants had significantly lower DI (Figure 6L), less *Rc* biomass (Figure 6M), and decreased H_2_O_2_ contents (Figure S13C). These results suggest that *TaRBOH3* negatively regulates SE resistance, possibly through mediating ROS in wheat.

TaRHC1A, as a RING‐type E3 ligase, possibly mediates TaHDA9 degradation through ubiquitination. Immunoblotting results using an anti‐GST antibody showed that the level of TaHDA9 protein was decreased sharply in *TaRHC1A*‐OE plants compared with control after 60 min. However, adding MG132 significantly inhibited the decrease of TaHDA9 (Figure S13D). Immunoblotting results from co‐expression of 35S::TaHDA9‐Flag‐GFP and 35S::TaRHC1A‐His‐GFP in *N. benthamiana* also indicated that TaRHC1A significantly decreased TaHDA9 protein abundance through the 26S proteasome pathway (Figure S13E). These results showed that TaRHC1A reciprocally mediates TaHDA9 degradation through ubiquitination.

### RcPL3 enhances the deacetylation of TaRHC1A by TaHDA9

To test whether RcPL3 affects TaHDA9 deacetylating TaRHC1A, we transiently co‐expressed vectors 35S::TaRHC1A‐Flag‐GFP and 35S::TaHDA9‐His‐GFP with or without 35S::RcPL3‐His‐GFP into *N. benthamiana* leaves. Immunoblotting results indicated that the addition of RcPL3 significantly reduced the Kac and protein level of TaRHC1A compared with controls (Figure 6N). Subsequently, we extracted proteins from *RcPL3*‐OE lines and controls. Immunoblotting results with anti‐TaRHC1A specific antibody indicated that TaRHC1A exhibited decreased protein abundance and reduced Kac level in *RcPL3*‐OE lines compared with controls (Figure 6O). Furthermore, immunoblotting results using an anti‐TaRBOH3 specific antibody showed that TaRBOH3 had higher protein abundance in the *RcPL3*‐OE and *TaHDA9*‐OE lines than in their controls (Figure 6P,Q). However, Y2H assays indicated that RcPL3 did not directly interact with TaRHC1A (Figure S14) but strongly interacted with TaHDA9 (Figure 4A−D), implying that RcPL3 indirectly influences TaRHC1A by mediating TaHDA9. These results suggested that RcPL3 reduced the accumulation of TaRHC1A protein, possibly by promoting TaHDA9‐mediated deacetylation (Figure 6R).

### HY‐19741 exhibits antifungal activity

Given that RcPL3, as a virulent effector, promotes *Rc* invasion into wheat plants by mediating TaHDA9‐TaRHC1A‐TaRBOH3 pathway, we predicted RcPL3‐binding compounds in the FDA‐approved drug libraries to identify the antifungal candidates for *Rc* prevention in wheat according to the virtual screening pipeline (Figure 7A). After the Vina value calculation, a total of 26,680 available compounds with low molecular weight were identified for further analysis. After three rounds of docking, the predicted max affinity of 267 candidates ranged from −8.08 to −10.32 kcal/mol (Table S14). Based on the threshold of −10.00 kcal/mol, the top 10 compounds were selected, and 5 of them with relatively low costs were subsequently tested by agar diffusion assays. Results showed that adding HY‐19741 (CAS:1430844‐80‐6) could significantly inhibited the *Rc* growth on the PDA medium, but adding any other compound does not always significantly inhibit the growth of *Rc* at all time points (Figures 7B−G and S15A−D). Prediction showed that HY‐19741 potentially interacted with RcPL3 (Figure 7H), and thereby might weaken the interaction of RcPL3 with TaHDA9. Furthermore, competitive pull‐down assays showed that the presence of HY‐19741 obviously reduced the interaction between RcPL3 and TaHDA9 (Figure 7I).

**FIGURE 7:**
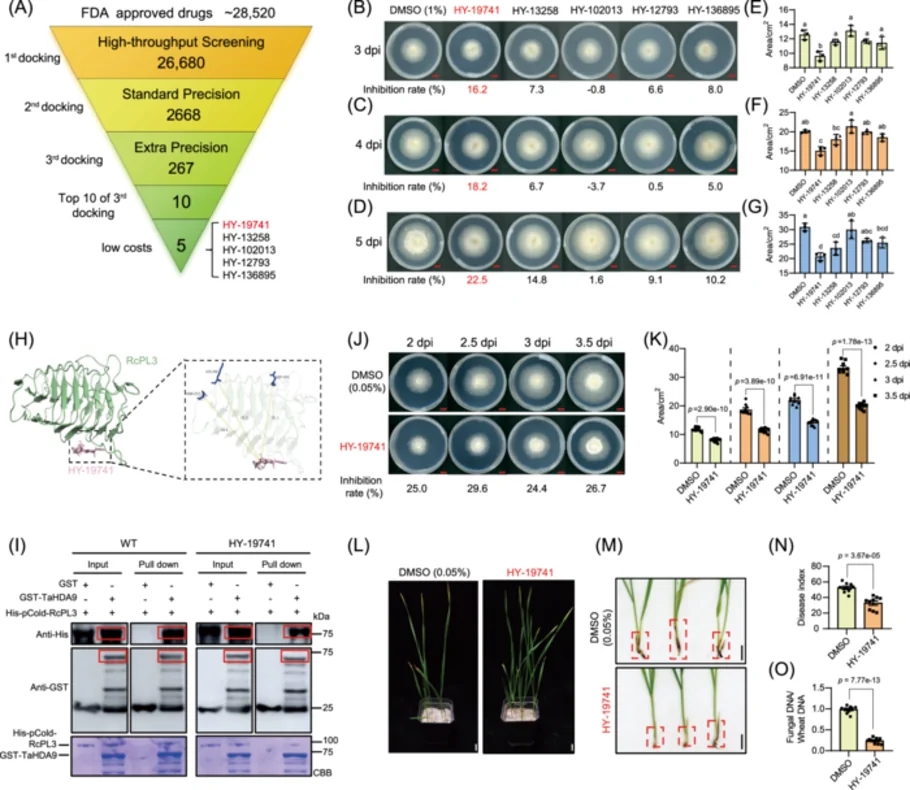
Antagonist HY‐19741 prevents the RcPL3‐mediated *Rc* infection. (A) Identification of the candidate compounds inhibits RcPL3 by virtual screening. A total of 28,520 approved drugs (compounds) were selected for docking with RcPL3 using Autodock vina. First, conduct a high‐throughput virtual screening (HTVS) with an exhaustiveness level of 8 for all available compounds and select the top 10% with the highest scores to move to the next round. Secondly, conduct a more precise docking (standard precision screening, SP) (exhaustiveness: 16) on the compounds and select the top 10% with the highest scores for the final assessment (extra precision screening, XP) with an exhaustiveness level of 128. (B−D) Images and the inhibition rates of *Rc* treated with 50 μM HY‐19741, HY‐13258, HY‐102013, HY‐12793, and HY‐136895 at 3 (B), 4 (C), and 5 dpi (D), respectively. (E−G) Analysis of variance of mycelium growth area treated with above five candidate compounds at 3 (E), 4 (F), and 5 dpi (G), respectively. (H) Three‐dimensional interaction pattern of RcPL3 and HY‐19741. Yellow dotted lines, pink stick, and green cartoon indicate hydrogen‐bond interactions, HY‐19741, and RcPL3, respectively. (I) The competitive pull‐down assay verifies that the presence of HY‐19741 obviously weakens the interaction between RcPL3 and TaHDA9. The red boxes indicate RcPL3 and TaHDA9. (J) Images and the inhibition rates of *Rc* treated with 50 μM HY‐19741 at 2, 2.5, 3, and 3.5 dpi, respectively. (K) Analysis of variance of mycelium growth area treated with HY‐19741 at the 2−3.5 dpi. (L−O) HY‐19741 prevents *Rc* infection in the pot experiment. Phenotypes of plants (L and M), sharp eyespot DI (N), and *Rc* biomass (O) treated with 50 μM exogenous HY‐19741. Scale bars, 1 cm in (B−D, J, and M) and 2 cm in (L). Data in (E−G) are presented as the mean values ± SE with individual data point; Different letters indicate significant differences (*p* < 0.05), as determined by one‐way ANOVA with Tukey's multiple comparisons test. Data in (K) and (N, O) are presented as the mean ± SE with individual data points (Unpaired two‐sided *t*‐test with at least three biologically independent replicates). In (B−D), (I, J), and (L, M), the experiments were conducted with at least three biological replicates showing similar results.

Treatment with the decrease of dimethylsulfoxide (DMSO) demonstrated that adding HY‐19741 effectively inhibited fungal growth of *Rc* (Figure 7J,K). Meanwhile, exogenous HY‐19741 enhanced SE resistance in wheat plants (Figure 7L−O). Moreover, immunoblotting results using gene‐specific antibodies revealed that the addition of HY19741 significantly decreased the abundances of endogenous TaHDA9 (Figure S16A) and TaRBOH3 (Figure S16B) proteins, but significantly increased the abundance of endogenous TaRHC1A (Figure S16C) in wheat plants. We further test the expression levels of ROS‐related genes after adding HY‐19741. qRT‐PCR results revealed that the expression levels of four ROS‐related genes (*TaPOD1*, *TaPOD3*, *TaSOD1*, and *TaAPX2*) were significantly up‐regulated after adding HY‐19741 (Figure S16D−G). Taken together, these results suggest that HY‐19741 prevents damage of SE in wheat, possibly by mediating the TaHDA9‐TaRHC1A‐TaRBOH3 model.

In summary, we sequenced five new high‐quality de novo reference genomes of *Rc* and established a comprehensive genome‐wide variation atlas for this pathogen. We further identified RcPL3 as a key effector to recruit TaHDA9 that could suppress TaRHC1A by deacetylation and thus inhibited degradation of TaRBOH3 by TaRHC1A, finally resulting in ROS burst that contributed to SE susceptibility in wheat plants. However, as an antagonist, HY‐19741 attenuates *Rc* infection by mediating the TaHDA9‐TaRHC1A‐TaRBOH3 cascade, thereby modulating ROS accumulation in wheat.

## DISCUSSION

SE is a devastating disease that affects the yield and quality of wheat worldwide [48]. Plants infected by *Rc* exhibit dark‐bordered lesions on the stem bases, leading to breakage at the internodes and premature spike senescence or white heads [28]. In recent years, the severity of SE in wheat production areas of China has escalated due to large‐scale application of straw retention, high sowing density, heavy use of nitrogen fertilizers, and widespread planting of susceptible cultivars. In 2024, SE affected about eight million hectares of wheat in China [49]. SE is estimated to cause economic losses of more than 15 million US dollars annually in China [1]. Fungicide applications by seed coating and spraying provide partial control, but there are ongoing concerns regarding fungicide resistance, environmental pollution, and cost [4, 5]. SE‐resistant wheat is the preferred alternative, but the available sources of resistance are poorly defined and provide limited protection [50, 51]. Therefore, the large‐scale collection of *Rc* isolates and genome sequencing of dominant isolates will contribute to a better understanding of genomic characteristics and pathogenicity of *Rc*, and also provide information to assist the development of resistant wheat cultivars.

*Rc* represents an important Basidiomycete pathogen of cereals, and among them, the genome sequences of 42 *Rs* isolates, 21 *Pst* isolates, and 37 *U. maydis* are listed by NCBI (https://www.ncbi.nlm.nih.gov/datasets/genome). On this basis, Wang et al. [52] yielded the pan‐genome of *Pst* through integrating FH11 genome, which provided a foundation for further research on pathogenicity mechanisms of the wheat stripe rust pathogen *Pst*. However, the genomic information of *Rc* and our understanding of its pathogenic mechanisms are limited. To elucidate the *Rc* genetic variation and molecular mechanism of pathogenicity, we sequenced genomes of five *Rc* isolates and re‐sequenced the genomes of 207 others. The Hi‐C assembled genomes were 42.53–49.34 Mb, and their N50 scaffolds were 3.85–4.70 Mb (Table S3). We assigned averaged 93.54% of assembled sequences to 12 chromosomes (Table S3 and Figure 2A−D). Additionally, the 12 chromosomal pseudomolecules showed high collinearity with Hi‐C assembled sequences among isolates R0301, R5‐3, and R1‐8 (Figure 2E,M and S2). These results indicated that we constructed five high‐quality *Rc* genomes. Resequencing provided the evidences of geographical distribution of related *Rc* isolates from Sichuan, Shaanxi, and Xinyang in Henan; Hubei and Zhejiang; and Shandong, Jiangsu, Anhui, and Henan (except Xinyang), forming different clusters (Figure 2K).

Previous studies showed that small secreted effector proteins encoded by plant necrotrophic pathogens play important roles in fungal pathogenicity [18, 53, 54]. For example, *P. nodorum* secretes necrotrophic effectors (e.g., SnToxA, SnTox267, SnTox3, and SnTox5) that target specific host susceptibility genes to trigger PCD, which supports pathogen nutrition and growth [54]. Similarly, *F. graminearum* secrets an orphan effector protein Osp24 into plant cells to cause proteasomal degradation of TaSnRK1α and promotes host susceptibility [19]. The plant pathogen *C. fructicola* secretes effector CfEC12, which contains a CFEM (common in several fungal extracellular membrane proteins) domain. CfEC12 targets NPR1 regulator NIMIN2, preventing the interaction between NIMIN2 and NPR1 and reducing NPR1‐depending defense responses during *C. fructicola* infection [55]. In addition, the rice blast pathogen *M. oryzae* secretes effector MoSPAB1 that directly activates rice *Bsr‐d1* expression to facilitate pathogenesis [56]. In this study, we constructed a *Rc* pan‐secretome of 506 effectors and found that 43 effectors were upregulated during *Rc* infection, possibly affecting pathogenicity. We further verified that RcPL3 could be involved in pathogenicity by multiple strategies. BLASTp analyses revealed that the homologs of RcPL3 can be found only in *Rhizoctonia* fungi (Table S15), indicating that a *Rhizoctonia*‐specific protein.

As common wheat is allohexaploid (AABBDD) with the large genome size (~16 Gb) [57], it is difficult to directly clone regulatory genes affecting host response to SE by a traditional forward genetic strategy. With the sharp improvement of wheat genomes [57, 58] and rapid development of multi‐omics technologies [59], a number of genes affecting SE response have been cloned by integrating transcriptomic data with other molecular techniques [60, 61, 62]. However, the molecular mechanisms of most genes affecting SE response are still unclear, and applications in breeding for resistance are very limited. Screening susceptibility factors targeted by *Rc* effectors and understanding the pathogenic mechanisms could provide novel strategies for achieving SE resistance by breeding. In this study, we used the effector RcPL3 as bait to screen histone deacetylases. The Kac of lysine residues as a reversible protein post‐translation modification was reported to have a role in plant immunity [63]. Recent proteomic analyses have revealed that non‐histone proteins are often acetylated and contribute to key cellular processes related to physiology and disease response [40, 64]. HDA9 was reported to interact with and deacetylates non‐histone protein WRKY53 to function in repressing plant response to salt stress in *Arabidopsis* [65]. In the study, we confirmed that TaHDA9 negatively modulated SE response, possibly due to its deacetylation of TaRHC1A, hence providing potential candidate genes for creating SE‐resistant germplasms by gene editing techniques.

TaRHC1A encodes a RING‐type E3 ligase. Previous studies showed that E3 ligases have integral roles in plant disease resistance [46, 66]. In *Arabidopsis*, the E3 ligase ATL1 positively regulated powdery mildew resistance, and EDR1 negatively regulated ATL1 activity to suppress cell death [67]. A microtubule‐associated C4H3‐type E3 ligase ubiquitinated and promoted degradation of serine hydroxymethyltransferase1 (SHMT1), initiating plant immune signaling to confer broad‐spectrum resistance against pathogens [68]. In rice, a RING‐H2 finger E3 ubiquitin ligase (OsRFPH2‐10) caused antiviral defense at an early stage of infection by reducing *dwarf virus* P2 protein level [69]. In the present study, we found a new E3 ligase, TaRHC1A, positively regulating SE resistance by promoting the degradation of TaRBOH3 (a wheat ROS synthase). The reduced protein level of TaRHC1A deacetylated by TaHDA9 was due to recruitment by RcPL3, causing ROS accumulation to infection.

ROS are important components of the plant immune system [20]. High ROS levels induce cell death, causing a hypersensitive response (HR). Cell death often increases plant resistance to biotrophic pathogens, but contributes to infection by necrotrophic and hemi‐biotrophic pathogens [70, 71]. For instance, *B. cinerea* and *S. sclerotiorum* utilized plant HR for rapid colonization of their hosts [70]. Local accumulation of ROS in infected cells of an *Osmore1a* mutant led to rapid HR‐induced cell death, facilitating infection by *C. miyabeanus* in rice [71]. In wheat, the accumulation of ROS promoted infection by *Rc*, while ROS scavenging enhanced resistance to it. In this work, we confirmed a RcPL3‐TaHDA9‐TaRHC1A‐TaRBOH3 model for the modulation of resistance to *Rc* in wheat by controlling ROS accumulation and *RcPL3*‐mediated *Rc* infection can be disrupted by HY‐19741, which identified potentially valuable genes and fungistatic compounds for reducing SE by breeding.

## CONCLUSION

The study sequenced five *Rc* genomes and, following large‐scale genome resequencing, constructed a variation map of *Rc*. We further revealed pathogenicity mechanism of *Rc* through which effector RcPL3 recruited TaHDA9 to inhibit TaRHC1A, allowing ROS accumulation aiding *Rc* infection of wheat plants. Moreover, we demonstrate a strategy for SE disease prevention by antagonist HY‐19741. However, some limitations of our study should be kept in mind when interpreting our results. First, we constructed high‐quality genome maps and identified a key virulence effector *RcPL3* in *R. cerealis*. Since there is a lack of effective approaches to generate *Rc* mutants so far, we only verified the function of *RcPL3* through VIGS (virus‐induced gene silencing) and overexpression in wheat. However, generating knockout mutants of *RcPL3* could provide more direct evidence, in future work, to create *Rc* isolates with the genetic background of non‐homologous end‐joining deficiency on purpose of improving the probability of obtaining *RcPL3* mutants. Second, the contributions of three homoeologous genes were not fully dissected in wheat. Finally, the target specificity of the compound HY‐19741 has not been definitively established and it needs more data to support.

## METHODS

### Sampling and isolation of pure fungal cultures

Wheat plants with SE symptom were sampled from wheat fields at the 30 locations (Table S1) covering almost all major growing regions in China between April 1 and mid‐June from 2014 to 2020 (Figure 1A,B). At each location, 1−7 wheat fields with a distance of at least 2 km between neighboring fields were sampled. In each field, tissues from at two diseased plants separated by at least 200 m were collected. After disinfection, the collected tissue for each sample was put on potato‐dextrose agar (PDA) and incubated at 25°C in darkness 3−4 days. Pure isolates were made from diseased tissues by the single hyphal tip method. All isolated cultures were maintained on PDA plates at 4°C.

### Pathogen culture, fungal DNA extraction, and rDNA‐ITS sequence analysis

The pure fungal cultures were inoculated on PDA and covered with sterile cellophane for 7−10 d at 25°C in darkness. The cultured mycelium was dried at room temperature and transferred to 1.5 mL centrifuge tubes using forceps. Genomic DNA was extracted from the fungal isolates using an Ezup Column Fungi Genomic DNA Purification Kit following the manufacturer's instructions (B518259, Sangon Biotech).

The ITS regions were amplified by primers ITS1 and ITS4 [72]. The PCR amplification products were sequenced in both directions after selecting band sizes by Ren et al. [73]. The PCR amplification products were sequenced in both directions after selecting band sizes by 1.5% agarose gel electrophoresis. If sequencing of the PCR products showed that they were not a single sequence, the PCR products were ligated with the pMD19‐T vector (3271, TaKaRa) and transformed into and amplified in *Escherichia coli* DH5a (DL1001, WeiDi). Plasmids with at least 20 recombinant *E. coli* colonies after verification of band size by PCR amplification were selected randomly for sequencing sub‐clones.

### Phylogenetic analyses of the ITS sequences

All 852 ITS sequences obtained in this study and 59 available ITS sequences from 22 binucleate *Rhizoctonia* (BNR) AGs (Table S1), including AG‐A, AG‐Ba, AG‐Bb, AG‐B(o), AG‐C, AG‐DI, AG‐DII, AG‐DIII, AG‐E, AG‐Fa, AG‐Fb, AG‐G, AG‐H, AG‐I, AG‐K, AG‐L, AG‐O, AG‐P, AG‐Q, AG‐R, AG‐S, and AG‐U from GenBank were assembled to construct a neighbor‐joining tree using Phylip software [74], and a phylogenetic tree was displayed by iTOL [75].

### Evaluation of *Rc* isolate pathogenicity

Four wheat cultivars, Chinese Spring (CS) and Yumai 49 (YM49), Zhoumai 18 (ZM18), and Aikang 58 (AK58) were used for pathogenicity tests of each *Rc* isolate. The experiment was carried out in plastic pots of 7 × 7 × 7 cm. Wheat seeds were sterilized with 95% ethyl alcohol for 3 min and then rinsed in sterile water three times. Soil for planting was autoclaved at 121°C for 1 h at 0.1 MPa. When soiling, 170 g sterilized soil containing 0.8 g colonized wheat kernels was placed into a plastic pot, followed by placing 16 sterilized seed that were then covered with 30 g of sterilized soil. Three replicates were performed for each cultivar. Twenty‐four plastic pots were placed onto one tray, including one susceptible cultivar YM49 as the control. All plastic pots were watered from the bottom of the trays.

SE was evaluated about 28 dpi, using a 0 (no symptoms) to 9 (complete necrotic damage) scale following the previous method [28]. Briefly, level 0, plant growth without infection symptom is healthy; level 1, the first leaf sheath of the plant turns brown or slight SE lesions appears; level 3, the first leaf sheath of the plant has obvious SE lesions; level 5, the second leaf sheath of the plant has obvious SE lesions; level 7, the third leaf sheath of the plant has obvious SE lesions; level 9, complete necrotic damage. DI was calculated for each replicate using 12 test disease seedlings according to the formula DI (%) = Σ (*f_i_* × *y_i_*) × 100/(Σ *f_i_* × 9), where *y_i_* is the disease score for each plant and *f_i_* is the number of plants with each score. At least three independent biological repeats were performed.

### Genome sequencing and assembly

We chose five representative *Rc* isolates (R0301, R1‐6, R1‐8, R5‐3, and R10‐1) from AG‐DI subgroup for genome sequencing by PacBio Sequel II. These five isolates covered with sterile cellophane were grown in PDA at 25°C in darkness for 4−10 days. Mycelium of each isolate was transferred into 1.5 mL centrifuge tubes using forceps, frozen in liquid N_2_, and freeze‐dried. After extraction using a cetyltrimethylammonium bromide (CTAB) method [76]. Library construction was performed according to the standard protocol provided by PacBio, including sample quality inspection, library preparation, library quality assessment and sequencing. A total of 3.72 Gb of clean CCS data was ultimately retained after filtering across five sequenced isolates. Hi‐C sequencing was further performed on three isolates (R0301, R1‐8, and R5‐3). Hi‐C libraries were created using a previously described method [77]. Three Hi‐C fragment libraries with fragment sizes of 300−700 bp were constructed and sequenced on the Illumina platform. A total of 149,921,294 paired reads were generated.

The filtered CCS data were assembled using Hifiasm software [78]. Pilon software [79] was used to further correct errors using second‐generation data, and a more accurate genome was obtained. The corrected contigs were assembled into scaffolds by LACHESIS [80]. Adjacent contigs were linked together by filling the gap with “N.” Thirty‐six to 63 sequences were anchored and oriented onto a twelve chromosome‐scale super scaffold.

### Genome annotation

A combination of de novo and structure prediction strategies was used to identify and annotate repeat sequences in the *Rc* genome. LTR_FINDER (version 1.05) [81] and RepeatScout (version 1.0.5) [82] were used for ab initio prediction. The identified repeats were compared for classification into repeat categories using PASTEClassifier [83]. Then, it was combined with the Repbase database [84] as the final repeat sequence database. RepeatMasker (version 4.0.6) software was used to predict repeat sequences in *Rc* based on the established repeat sequence database [85].

Protein‐coding genes were predicted by integrating results from ab initio predictions, protein homology, and RNA‐seq read mapping. For ab initio prediction, we used gene predictor software Genscan [86], Augustus (version 2.4) [87], GlimmerHMM (version 3.0.4) [88], GeneID (version 1.4) [89], and SNAP (version 2006‐07‐28) [90]. Predicted proteins from two genomes (*Ceratobasidium theobromae* and *Rhizoctonia solani*) were used to perform homology‐based prediction using GeMoMa63 (version 1.3.1) [91]. To facilitate gene annotation, transcriptomic datasets were generated from vegetative hyphal samples of five isolates (R0301, R1‐6, R1‐8, R5‐3, and R10‐1) by Illumina RNA‐seq. Hisat2 (version 2.0.4) and Stringtie (version 1.2.3) [92] were used for assembly based on reference transcripts, and TransDecoder (version 2.0) [93] was used for prediction of Unigene sequences based on the transcriptome assembly using PASA (version 2.0.2) [94]. Finally, EVM (version 1.1.1) [95] was used to integrate the prediction results from the above three methods, and PASA (version 2.0.2) was used for modification. A total of 10,697−12,296 protein‐coding genes were identified in the 5 *Rc* genomes.

Functional annotations were assigned by aligning the *Rc* protein sequences to various databases including KOG [96], KEGG [97], Swiss‐Prot [98], TrEMBL [98], Nr [99], CAZy [100], and PHI‐base [101] using Diamond (v0.9.29.130) [102], E‐value ≤ 1e − 5). Gene Ontology (GO) annotations were assigned by Blast2GO software [103]. ORF domains were found in the Pfam database [104] using hmmer software [105]. Fungal effector proteins were predicted based on Effector P software [106].

### Comparative genome analyses

Protein sequences from six *Basidiomycotina* genomes: *R. solani* AG‐1 IA (GenBank: GCA_016906535.1), *Botryobasidium botryosum* FD‐172 SS1 (GenBank: GCA_000697705.1), *Moniliophthora roreri* MCA 2952 (GenBank: GCA_001466705.1), *Postia placenta* MAD‐698‐R‐SB12 (GenBank: GCA_002117355.1), *Puccinia striiformis* 93‐210 (GenBank: GCA_002920065.1), *Ustilago maydis* 521 (GenBank: GCF_000328475.2), and one *Ascomycotina* genome (*Pyricularia oryzae* MZ5‐1‐6) (GenBank: GCA_004346965.1) were obtained. Orthologous groups (gene families) among these genomes and the five sequenced *Rc* genomes in the study were identified using OrthoFinder software [30]. The core and dispensable gene families, as well as the single‐copy gene families of each pathogen, were then statistically analyzed. The results of this analysis were used to construct a flower diagram, and the function of gene families was annotated using Pfam database.

A phylogenetic tree was constructed using the maximum likelihood method (ML). Divergence times were estimated under a relaxed clock model using MCMCtree in PAML (version 4.9i) [107] with the following parameters: burnin = 1,000,000, nsample = 20,000, and sampfreq = 500, and divergence dates for *M. roreri* (227–277 MYA), *P. placenta* (224–313 MYA), *P. striiformis* (447–616 MYA), *U. maydis* (415–482 MYA), and *P. oryzae* (626–806 MYA) obtained from Timetree [108] were further used to calibrate the divergence time. Evolutionary analysis of gene synteny and collinearity was performed using MCScanX [109], and syntenic gene pairs were visualized using the dotplot script in the JCVI package (https://github.com/tanghaibao/jcvi). CAFE (version 4.2) (parameters: lambda‐s) [110] was used to identify the expansion and contraction of gene families in the *Rc* genome relative to other fungal species.

### Sample preparation and transcriptome analysis

The wheat cultivar YM49, highly susceptible to SE, was inoculated with *Rc* (R0301) at the two‐leaf stage in the greenhouse, and diseased stems at 3 dpi, 5 dpi, and 7 dpi, and in vitro mycelia were collected. Each sampling had three independent biological replicates. Sampled stems were rapidly frozen in liquid nitrogen and stored at −80°C for RNA sequencing. Stems of line *TaRHC1A*‐OE#5 and Fielder at the heading stage in a greenhouse were sampled for transcriptome analysis.

RNA concentration and purity were measured using NanoDrop 2000 (N208, Thermo Fisher Scientific). RNA integrity was assessed using an RNA Nano 6000 Assay Kit (5067‐1511, Agilent) on an Agilent 2100 Bioanalyzer (G2939BA, Agilent). Clustering of the index‐coded samples was performed on a cBot Cluster Generation System using a TruSeq PE Cluster Kit (v4‐cBot‐HS (Illumina)) according to the instructions. After cluster generation, the library preparations were sequenced on an Illumina platform and paired‐end reads were generated.

Raw data (raw reads) in fastq format were first processed through in‐house Perl scripts. In this step, clean reads were obtained from the raw data by removing low‐quality reads and reads containing adapter residues or ploy‐N. At the same time, Q20, Q30, GC‐content, and sequence duplication levels of the clean data were calculated. All downstream analyses were based on clean data with high quality. Hisat2 (version 2.0.4) [111] was used to map the clean read data to the reference *Rc* genome (R0301) for this study or to the Chinese Spring (CS) wheat genome published by the International Wheat Genome Sequencing Consortium (IWGS, RefSeq. v1.0). The reads were reassembled using StringTie (version 1.2.3) [92]. Gene expression levels were estimated by fragments per kilobase of transcript per million fragments mapped (FPKM). Differential expression analysis of two conditions/groups was performed using DESeq2, which provides a statistical routine for determining differential expression in digital gene expression data using a negative binomial distribution model. The resulting *p* values were adjusted using the Benjamini‐Hochberg approach for controlling the false discovery rate. *Rc* genes with an adjusted *p*‐value < 0.05 found by DESeq2 were assigned as differentially expressed, whereas wheat genes with an adjusted *p*‐value < 0.01 were assigned as differentially expressed. DEGs were identified by separately comparing each inoculation time‑point (3 dpi, 5 dpi, and 7 dpi) against the uninoculated control (0 dpi, in vitro mycelia).

### Genomic resequencing and variant calling

To identify genomic variation in *Rc*, we carried out genome resequencing of 207 *Rc* isolates using an Illumina NovaSeq. 6000. DNA libraries (350 bp) for Illumina sequencing were constructed for each isolate according to the manufacturer's specifications. After DNA library construction, sequencing was performed on a NovaSeq. 6000 platform by Biomarker Technologies Co., Ltd. (Beijing), with 150‐bp read lengths. Raw reads were filtered based on the following criteria: pair‐end reads with >10% “N” bases; reads on which more than 50% of the bases had a quality score less than 20 (Phred‐like score); sequencing adapter. Finally, high‐quality sequences were obtained for subsequent analyses. All clean reads for each isolate were mapped to the R0301 genome using the MEM algorithm in Burrows–Wheeler Aligner (bwa‐mem2 version 2.2) [112]. The mapping results were sorted, and duplicate reads were removed using Samtools (version 1.7) [113]. Genetic variants including SNPs and Indels (short insertions and deletions) were detected using Genome Analysis Toolkit (GATK) [114]. SNPs identified by GATK were further filtered with only SNPs with QUAL ≥ 30, QD ≥ 2.0, MQ ≥ 40, and FS < 60 considered as high‐quality SNPs. Finally, 719,791 high‐quality SNPs were obtained. SNP annotation was performed on the basis of the R0301 genome using snpEff software [115], and SNPs were categorized into intergenic regions, upstream or downstream regions, and exons or introns. SNPs in coding exons were further classified as synonymous or nonsynonymous. InDels in exons were grouped according to whether they led to a frameshift.

### Population genetic diversity and structure analysis

To analyze the phylogenetic relationships of the 207 isolates, we constructed a phylogenetic tree using the neighbor‐joining method with the Kimura 2‐parameter/p‐distance model in MEGA‐CC software (MEGAX) [116], with 1000 bootstrap replicates.

In total, 261,329 high‐confidence SNPs were used to infer the population structure of 207 isolates using ADMIXTURE (version 1.22) [117], with *K* values (the putative number of populations) ranging from 1 to 10. We assessed the number of sub‐populations using five‐fold cross‐validation. The *Q* matrix with each *K* value stacked as a bar plot was generated using the R package Pophelper [118].

PCA of the SNPs was performed using the smartPCA program from the EIGENSOFT package using the default parameters [119]. The first (two) PCs were used to visualize the grouping of samples, explaining 13.41% (2.53%) of the total variation.

For LD analysis, complete and partial LD were calculated between each pair of SNPs using PLINK software (version 1.9) [120]. The squared correlation coefficient (*r* ^*2*^) values were analyzed for all chromosomes with a 1000‐kb window. LD blocks were defined with the following parameters: –ld‐window‐r20–ld‐window 99999 –ld‐window‐kb 1000. π and *F*_*ST*_ were calculated using vcftools [121] based on the high‐confidence filtered SNPs. The π value for each SNP was calculated, and nucleotide diversity level was measured using a 100‐kb window with a step size of 10 kb for each sub‐population.

### Genome‐wide association study (GWAS) analysis

A total 261,329 SNPs with minor allele frequency >0.05 and a missing data rate of 0.8 or less across the entire population were used for GWAS. A kinship (K) matrix generated with the FaST‐LMM program (version 2.07.20140723) [122] was used to correct the population structure. GWAS analyses were performed using the linear mixed model algorithm implemented in the FaST‐LMM program. The modified Bonferroni correction was used to determine the genome‐wide significance thresholds of the GWAS, corresponding raw *p* values of 1.0e‐05, or −log_10_ (*p*) values of 5, respectively.

### Quantitative real‐time PCR (qRT‐PCR) analysis post R0301 infection

Based on the results of effector prediction, transcriptome analysis, and GWAS, we selected the key effector gene *RcPL3* (Gene ID: EVM0004929) for qRT‐PCR verification. Primer 5.0 was used to design specific primers. RNA was extracted using a Plant Total RNA Extraction Kit (R019‐50, GeneBetter Biotech) according to the manufacturer's specifications. cDNA was reverse‐transcribed using a PrimeScript RT Reagent Kit with gDNA Eraser (RT‐01032, FORE GENE) following the manufacturer's instructions. qRT‐PCR was performed on a Bio‐RadCFX Manager 3.1 Sequence Detection System with three biological replicates using specific primers (Table S16). Each reaction contained 2 µL of cDNA, 0.4 µL of each primer (forward and reverse), 10 µL of SYBR Premix ExTaq II (R601, NOBELAB), and 7.2 µL of nuclease‐free water. The detailed procedure for qRT‐PCR followed Zhang et al. [123, 124] and Hou et al. [125]. The *β*‐actin gene (GenBank: AB181991) was used as the internal control.

### Barley stripe mosaic virus (BSMV)‐mediated gene silencing

Yumai 49, with high susceptibility to BSMV, was used to conduct BSMV‐induced gene silencing assays. The primers for *RcPL3* (EVM0004929) were generated from 222‐bp cDNA fragments using Primer 3.0 (Table S16). The *α*, *β*, and *γ* RNAs of the BSMV genome were synthesized from linearized plasmids, using RiboMAX^TM^ Large Scale RNA Production System‐T7 (P1300, Promega, USA) and Ribo m7G Cap Analog (P1712, Promega). Transcripts of each vector (*α*, *β*, *γ*, or recombinant γ‐gene) were mixed in a 1:1:1 ratio, and a 15 μL volume was added to 540 μL of FES buffer. Plants infected with virus were then grown at 24°C. Plants inoculated with BSMV:TaPDS (phytoene desaturase, GenBank: FJ517553.1) [126] were used as an index of BSMV infection. The BSMV:00 or BSMV:RcOSP1 were used as negative and positive controls, respectively. After silencing for 3 days, the plants were inoculated with *Rc*. About 30 infected stems were identified for SE response after 15 days. RNA was extracted from 5 stems for assessment of silencing efficiency.

To confirm function, we silenced genes *TaHDA9*, *TaRHC1A*, and *TaRBOH3* in wheat plants by VIGS assays. For silencing, 228‐bp, 182‐bp, and 300‐bp cDNA fragments from Chinese Spring were separately recombined to vector pSL038‐1 for VIGS. Vector linearization, transcription, and the virus mixture were performed as described above. The susceptible wheat accession Yumai 49 (average disease index (ADI) = 67.75) or moderately resistant wheat accession Pingyuan 50 (ADI = 38.57) were infected with the virus mixture at the two‐leaf stage and then inoculated with *Rc* after 3 days. Other procedures were the same as for *Rc* genes.

### Assays of signal peptide function in RcPL3

The predicted signal peptide (SP; 22 aa) of RcPL3 was cloned into the pSUC2 vector [127] that carries the yeast *SUC2* gene without an SP sequence. The resulting SP22‐SUC2 construct was transformed into the yeast *suc2* mutant YTK12 (YC1170S, WeiDi, China) [128] and assayed for growth on SD‐Trp‐glucose (0.074% tryptophan dropout supplement, 2% glucose, and 2% agar) and YPRAA (including YPR (yeast extract, peptone, raffinose) and Antimicyn A) (ZC1897, Zoman Biotechnology, China). YTK12 (YC1170S, WeiDi, China) transformants carrying the empty pSUC2 vector or pSUC2‐Avr1bSP were used as negative and positive controls, respectively. Invertase activity was detected by reduction of 2, 3, 5‐triphenyltetrazolium chloride (TTC, 0.1%) to insoluble red colored 1, 3, 5‐triphenylformazan (TPF).

### Assays for suppression of BAX‐induced cell death by RcPL3

Alleles *RcPL3* were separately cloned into pBWS and transformed into *A. tumefaciens* strain GV3101 (AC1001S, WeiDi, China) cultured in yeast extract peptone (YEP) broth medium with 50 mg/L rifampicin and 50 mg/L kanamycin. The *A. tumefaciens* resuspended to OD_600_ of 0.6 in infiltration solution (0.2 M morpholino ethane sulfonic (MES), 1 M MgCl_2_.6H_2_O, and 50 mM acetosyringone) was infiltrated into *N. benthamiana* leaves. At 24 h after infiltration of *N. benthamiana* leaves with *A. tumefaciens* carrying the empty GFP vector or 35S::RcPL3^ΔSP^‐Flag‐GFP construct, the same sites were infiltrated with *Agrobacterium* carrying the BAX construct. Plant cell death symptoms were examined after 4−5 days. *Agrobacterium* carrying the GFP vector was used as the negative control. Each infiltration experiment was repeated eight times. The proteins of all treatments were extracted and analyzed by immunoblotting with anti‐BAX antibody (A19684, Abclonal), anti‐Flag antibody (M20008, Abmart), and anti‐GFP antibody (AE012, Abclonal).

### Yeast two‐hybrid (Y2H) assays

The CDS of *RcPL3*, *TaHDA9*, and *TaRHC1A* were recombined to the vector pGBKT7 (V001, OEBiotech), respectively. The wheat plants of Jiyanmai 7 were used to construct a cDNA library of 0.8 million primary clones constructed by OEBiotech Co., Ltd. (China) for screening interaction proteins using the Y2H assay following the manufacturer's protocol. Yeast colonies that grew on SD‐Trp‐Leu‐His‐Ade and had β‐galactosidase activity were isolated as putative interaction colonies. After sequencing with primer T7, wheat genes corresponding to the inserts in these clones were identified by BLAST search. To directly assay their interactions, full‐length cDNAs or fragments of *TaHDA9*, *TaRHC1A*, and *TaRBOH3* were amplified and cloned into pGADT7 or pGBKT7 vectors. The SD/‐Trp/‐Leu transformants were isolated and assayed for growth on SD/‐Trp/‐Leu/‐His/‐Ade+AbA+X‐α‐Gal medium and filter lift assays were performed for galactosidase activity. The detailed procedure for Y2H followed Yang et al. [129].

### Split‐luciferase assay

The Split‐luciferase assay was performed as described by Zhao et al. [130] with minor modifications. The coding sequences of three genes (*RcPL3* from R0301, *TaHDA9* and *TaRHC1A* from Chinese Spring) were each cloned into pCAMBIA1300‐GWNLuc or pCAMBIA1300‐GWCLuc using a ClonExpress II One Step Cloning Kit (C112‐01, Vazyme, China). These constructs were transformed individually into *Agrobacteria* strain GV3101 (AC1001S, WeiDi, China). The *Agrobacteria* were collected and resuspended in infiltration buffer (0.2 M MES, 1 M MgCl_2_.6H_2_O, and 50 mM acetosyringone) to a final concentration of OD_600_ = 0.6. Pairwise combinations of the suspensions were infiltrated into young *N. benthamiana* leaves, which were placed in darkness for 12 h, followed by 2 days under 16/8 h light/darkness. Twelve‐sixteen leaves from 5 to 10 plants were collected and the abaxial side of each leaf was sprayed with 1 mM d‐Luciferin Potassium Salt (40902ES01, YEASEN), and placed in darkness for 10 min. Bioluminescence images captured using an Azure Sapphire Biomolecule Imager (AZI600‐01, Azure Biosystems) were converted to heatmaps using the 16‐color look‐up table (LUT) in ImageJ software.

### In vitro pull‐down assays and verification of key interaction sites between RcPL3 and TaHDA9

cDNA fragments of *RcPL3*, *TaHDA9*, *TaRHC1A*, and *TaRBOH3* were amplified and cloned into expression vectors pGEX6p‐1 and/or His‐pCold‐TF for purification of His‐pCold‐RcPL3, GST‐TaHDA9, GST‐TaRHC1A, His‐pCold‐TaRHC1A, and His‐pCold‐TaRBOH3 recombinant proteins. To assay their interactions, equal amounts of His‐pCold‐RcPL3 and GST‐TaHDA9, His‐pCold‐TaRHC1A and GST‐TaHDA9, or GST‐TaRHC1A and His‐pCold‐TaRBOH3, fusion proteins were incubated at 4°C for 2 h and mixed with glutathione resin (P2251, Beyotime) for affinity purification. The presence of His‐pCold‐RcPL3, His‐pCold‐TaRHC1A, or His‐pCold‐TaRBOH3 and empty GST in proteins eluted from glutathione resins was detected by Western blot analysis using anti‐His (1:5000 dilution, M20001, Abmart) and anti‐GST (1:5000 dilution, PTM‐5046, PTM Biolabs) antibodies. For assaying competition of His‐pCold‐RcPL3 vs GST‐TaHDA9, His‐pCold‐TaRHC1A versus GST‐TaHDA9, or GST‐TaRHC1A versus His‐pCold‐TaRBOH3, mixing equal amounts of His‐pCold‐RcPL3, His‐pCold‐TaRHC1A or His‐pCold‐TaRBOH3 with GST fusion proteins, respectively, were co‐incubated without/with GST‐TaHDA9 or GST‐TaRHC1A protein.

We created site‐directed mutagenesis of six mutants E94A (GAG‐GCG), D199A (GAC‐GCC), D266A (GAC‐GCC), R267A (CGC‐GCC), L268A (GTA‐GCA), and D266A(GAC‐GCC)/R267A(CGC‐GCC)/L268A(GTA‐GCA) within the GST‐TaHDA9 plasmid using a Fast Site‐Directed Mutagenesis Kit (KM101, Tiangen), respectively, as previously described [131]. PCR was conducted using site‐specific primers (Table S16). Mutants were verified by Sanger sequencing. His‐pCold‐RcPL3 with GST‐TaHDA9/GST‐TaHDA9 mutants (GST‐TaHDA9^D266A^, GST‐TaHDA9^D199A^, GST‐TaHDA9^L268A^, GST‐TaHDA9^E94A^, TaHDA9^R267A^, GST‐TaHDA9^D266A/R267A/L268A^) or GST proteins were expressed in *E. coli* BL21 (DE3). According to the instructions of the His‐tag Protein Purification Kit (P2226, Beyotime, China), His‐pCold‐RcPL3 was purified with buffer using BeyoGoldTM His‐tag purification resin. The pull‐down protein mixtures of GST‐TaHDA9/GST‐TaHDA9 mutant and GST were boiled with the SDS sample buffer, and the samples were detected by immunoblotting with anti‐GST antibody (PTM‐5046, PTM Biolabs) and anti‐His antibody (M20001, Abmart).

### Co‐immunoprecipitation (Co‐IP) assays

We extracted wheat proteins in *TaRHC1A*‐OE (with Flag tag) lines and the control (Fielder). TaRHC1A protein was IP with anti‐Flag antibody (M20008, Abmart) and Protein A/G PLUS‐Agarose (sc‐2003, Santa Cruz Biotechnology), and TaHDA9 was identified by anti‐TaHDA9 specific antibody (Abclonal). The eluted proteins were detected by immunoblotting with anti‐Flag antibody (M20008, Abmart) and anti‐TaHDA9‐specific antibody, respectively.

### Subcellular localization and bimolecular fluorescence complementation assay

For subcellular localization, CDSs of RcPL3, TaHDA9, and TaRHC1A were cloned into the pJIT163‐hGFP vector to generate Ubi::GFP‐RcPL3, Ubi::GFP‐TaHDA9, and Ubi::GFP‐TaRHC1A, respectively. The resulting constructs were transformed into wheat protoplasts of leaves (20%) and stems (80%) via polyethylene glycol (PEG)‐mediated transformation, respectively. After incubation for 14 h in the dark at 22−25°C, confocal microscopy (LSM980, Carl Zeiss) was used to examine the GFP fluorescence. To further investigate the impact of *Rc* infection on protein localization of TaHDA9, we re‐performed subcellular localization of TaHDA9 in wheat protoplasts. Briefly, the wheat protoplasts of seedling were extracted at 0, 1, 3, and 5 dpi with R0301, respectively. We then transformed the Ubi::GFP‐TaHDA9 vector into the protoplasts and examined the GFP fluorescence. For bimolecular fluorescence complementation (BiFC), the CDSs of RcPL3 and TaRHC1A were cloned into vectors pSAT1‐nEYFP‐C1, and the CDSs of TaHDA9 pSAT1‐cEYFP‐C1‐B to generate nEYFP‐RcPL3, nEYFP‐TaRHC1A, and cEYFP‐TaHDA9, respectively. The resulting constructs and empty vector were then co‐transformed into wheat protoplasts. After incubation for 20 h in the dark at 22−25°C, confocal microscopy was used to examine the YFP fluorescence.

### Ethyl methanesulfonate (EMS) mutants and overexpression and CRISPR‐Cas9 gene editing lines

Seeds of an EMS‐mutagenized tetraploid wheat cultivar Kronos mutant library were provided by J. Dubcovsky and colleagues, University of California, Davis. Two *TaHDA9‐2B* mutants (K733: A/G, splice acceptor variant; K2217: C/T, stop gained) and one *TaRHC1A‐3A* mutant (K3490: G/A, stop gained) identified in the library were twice backcrossed with WT and self‐pollinated to generate BC_2_ lines as previously described [132].

To obtain overexpression lines, we cloned the full‐length cDNA of *TaHDA9*, from Chinese Spring into the LGY‐OE3 vector containing a polyubiquitin gene promoter. The vectors containing the targeted genes were transformed by Agrobacterium‐mediated infection into immature embryos of wheat cultivar Fielder to obtain *TaHDA9*‐OE, *TaRHC1A*‐OE, and *TaHDA9* ^*R267A*^‐OE transgenic lines. For *TaHDA9*‐OE and *TaRHC1A*‐OE lines, positively T_0_ transgenic plants verified by PCR were self‐pollinated to T_2_ lines. For each gene, two or three independent T_2_ lines with high expression levels determined by qRT‐PCR were used in further analysis. For *TaHDA9* ^*R267A*^‐OE lines, positively T_0_ transgenic plants verified by PCR and qRT‐PCR were used in further analysis.

We created editing plants of *TaHDA9* in wheat using the CRISPR‐Cas9 system. To reduce potential off‐target effects, the target sites were selected using CRISPRdirect (http://crispr.dbcls.jp/) and CRISPOR (http://37.187.154.234/) based on homology searches against the wheat genome. Single‐guide RNAs (sgRNAs) targeting *TaHDA9* were designed using the Chinese Spring reference genome. The sgRNA fragments were amplified individually and cloned into the intermediate vector pMETaU6.1, and then were cloned into the pLGYE‐3 binary vector. After verification of the correct vector construction, genetic transformation was performed on Fielder to generate gene‐edited mutants.

*TaHDA9*‐Cas9‐mediated T_0_ plants were identified using Hi‐TOM sequencing, and were then self‐crossed into T_2_ lines. After further verification of the mutation sites through Sanger sequencing, the T_2_ edited lines were used for further analysis.

### Evaluation of the resistance to *Rc* in EMS mutant and transgenic wheat lines

The highly virulent *R. cerealis* (*Rc*) isolate R0301 was used to inoculate EMS and overexpression wheat plants. R0301 was routinely cultured on PDA at 25°C in darkness for 5 d, and wheat kernel inoculum prepared as previously described [28] was inoculated to seedlings grown in a greenhouse at 23°C with 16 h of light and 8 h of darkness. The SE symptoms recorded at 28 dpi were converted to DI scores. DNA from the pathogen‐inoculated stems was also extracted at 28 dpi for fungal biomass analysis. Relative fungal DNA biomass was calculated using the cycle threshold (Ct) value of *Rc* actin DNA (*RcActin*, GenBank No. KJ631110.1) against the Ct value of wheat genomic actin DNA (*TaActin*, GenBank No. AB181991.1) (Table S16) according to a previously described method [133, 134]. All experiments involved three biological replicates.

### Transient expression assays and immunoprecipitation

The recombinant plasmids 35S::RcPL3‐His‐GFP and 35S::TaHDA9‐Flag‐GFP were constructed, and transformed into Agrobacterium cells along with the control plasmid 35S::GFP for large‐scale expression. Bacterial cells were collected and resuspended in infiltration buffer (10 mM MgCl_2_, 10 mM MES‐KOH with pH 5.7, 150 µM acetosyringone). 35S::RcPL3‐His‐GFP + 35S::TaHDA9‐Flag‐GFP and 35S::TaHDA9‐Flag‐GFP + 35S::GFP were injected into leaf cells of 4‐week‐old N. benthamiana plants. After 72 h, total proteins were extracted from the N. benthamiana leaves using lysis buffer (20 mM Tris‐HCl, 2 mM DTT, 1% Triton X‐100, 800 μM PMSF, 250 mM sucrose; pH 8.0) containing the protease inhibitor cocktail according to our previous method [135]. Protein concentration was quantified using the Bradford assay with a standard curve, followed by Western blot analysis. The tagged proteins were detected using an anti‐Flag antibody (M20008, Abmart) and the internal reference protein was detected using an anti‐actin antibody (ABL1050, Abbkine).

35S::TaRHC1A‐Flag‐GFP + 35S::TaHDA9‐His‐GFP and 35S::TaRHC1A‐Flag‐GFP + 35S::GFP were injected into leaf cells of 4‐week‐old N. benthamiana plants by injection, and total proteins were extracted in the lysis buffer. TaRHC1A‐Flag‐GFP proteins from the above extracts were immunoprecipitated with anti‐Flag antibody (M20008, Abmart) and protein A/G PLUS‐Agarose (sc‐2003, Santa Cruz, USA) after a series of washing steps with phosphate‐buffered saline (137 mM NaCl, 2.7 mM KCl, 10 mM Na2HPO4, 2 mM KH2PO4, 0.2 mM DTT; pH 7.4) according to a previously reported method [135]. Eluted proteins were detected by immunoblotting with anti‐Flag antibody (M20008, Abmart, China) and pan‐anti‐acetylated lysine antibody (anti‐Kac antibody; 9681, Cell Signaling Technology). To determine whether TaHDA9 affects TaRHC1A expression, proteins from N. benthamiana leaves transiently expressing these constructs were extracted and analyzed through immunoblotting using the anti‐Flag antibody (M20008, Abmart). An anti‐actin antibody (ABL1050, Abbkine) was used as the loading control.

To investigate the effect of RcPL3 on the TaHDA9‐TaRHC1A model, 35S::TaRHC1A‐Flag‐GFP + 35S::TaHDA9‐His‐GFP + 35S::GFP and 35S::TaRHC1A‐Flag‐GFP + 35S::TaHDA9‐His‐GFP + 35S::RcPL3‐His‐GFP were introduced into N. benthamiana leaf cells by injection. Following protein extraction, TaRHC1A‐Flag‐GFP proteins were immunoprecipitated using anti‐Flag antibody (M20008, Abmart) and then detected by immunoblotting with anti‐Flag antibody (M20008, Abmart) and pan‐anti‐Kac antibody (9681, Cell Signaling Technology). To explore whether RcPL3 modulates TaRHC1A expression by TaHDA9, proteins from leaves transiently expressing 35S::TaRHC1A‐Flag‐GFP and 35S::TaHDA9‐His‐GFP, along with either 35S::RcPL3‐His‐GFP or 35S::GFP, were analyzed by immunoblotting using anti‐Flag antibody (M20008, Abmart), with anti‐actin antibody (ABL1050, Abbkine) serving as the loading control.

To compare the acetylation (Kac) levels of TaRHC1A‐Flag‐GFP and TaRHC1AK222R‐Flag‐GFP, 35S::TaRHC1A‐Flag‐GFP and 35S::TaRHC1AK222R‐Flag‐GFP were introduced into N. benthamiana leaf cells by injection, respectively. Following protein extraction, TaRHC1A‐Flag‐GFP proteins were immunoprecipitated using anti‐Flag antibody (M20008, Abmart, China) and then detected by immunoblotting with anti‐Flag antibody (M20008, Abmart) and pan‐anti‐Kac antibody (9681, Cell Signaling Technology). The proteins of N. benthamiana leaves transiently expressing 35S::TaHDA9‐Flag‐GFP and 35S::TaRHC1A‐His‐GFP (+/−10 μM 26S proteasome inhibitor MG132) or 35S::GFP were extracted and analyzed by immunoblotting with anti‐Flag antibody (M20008, Abmart). The anti‐actin antibody (A01050, Abbkine) was used as a loading control.

### Prokaryotic expression assays

GST‐TaHDA9, GST‐TaHDA9 mutants, GST‐TaRHC1A, His‐pCold‐RcPL3, His‐pCold‐TaHDA9, His‐pCold‐TaHDA9‐Flag, His‐pCold‐TaRHC1A‐Flag, His‐pCold‐TaRHC1A, His‐pCold‐TaRHC1A mutants, and His‐pCold‐TaRBOH3 recombinant constructs were generated by cloning the coding sequences into either the pGEX‐6P‐1 or pCold‐TF prokaryotic expression vector using the appropriate restriction enzymes (BamHI/NotI or NdeI/XhoI), respectively. Large‐scale expression of the above recombinant constructs was performed in *E. coli* BL21 (DE3) cells, and the proteins were purified using the GST‐tag Protein Purification Kit (P2262, Beyotime) or the His‐tag Protein Purification Kit (P2226, Beyotime). Immunoblotting experiments were conducted using the corresponding antibodies (Table S13).

### Nuclear‐cytoplasmic fractionation and immunoprecipitation

Total proteins of wheat seedlings were extracted in lysis buffer (10 mM Tris‐HCl with pH 8.0, 10 mM MgCl2, 0.1 mM PMSF, 0.4 M sucrose, and 5 mM β‐mercaptoethanol) with protease inhibitor cocktail according to the method previously described [136] with minor modification. The homogenate was filtered through a double layer of Miracloth. The flow‐through was spun at 1500 *g* for 20 min, and the supernatant, consisting of the cytoplasmic fraction, was centrifuged at 16,000 *g* for 20 min at 4°C and collected. The pellet was washed four times with 5 mL of nuclear resuspension buffer (0.25 M sucrose, 10 mM Tris‐HCl pH 8.0, 10 mM MgCl2, 1% TritonX‐100, 0.1 mM PMSF, 5 mM β‐mercaptoethanol) supplemented with protease inhibitor cocktail and then carefully overlaid on top of buffer C (1.7 M sucrose, 10 mM Tris‐HCl pH 8.0, 2 mM MgCl2, 0.15% TritonX‐100, 0.1 mM PMSF, 5 mM β‐mercaptoethanol) supplemented with protease inhibitor cocktail. These were centrifuged at 16,000 *g* for 60 min at 4°C. The final nuclear pellet was resuspended in nuclear lysis buffer (20 mM Tris‐HCl, pH 7.5, 20 mM KCl, 2 mM EDTA, 2.5 mM MgCl2, 20% glycerol, 250 mM sucrose, 5 mM DTT). As quality controls for the fractionation, Actin and histone H3 were used as cytoplasmic and nuclear markers, respectively.

To check how TaHDA9 protein accumulates in wheat seedlings after *Rc* infection, the nuclear and cytoplasmic proteins of wheat seedlings at 0, 1, 3, and 5 dpi, respectively, were extracted and analyzed through immunoblotting using the specific anti‐RcPL3 antibody and anti‐TaHDA9 antibody. The anti‐H3 antibody (Agrisera, Ca#AS10710) and anti‐actin antibody (ABL1050, Abbkine) were used as the loading control. Furthermore, TaHDA9 proteins were immunoprecipitated using anti‐TaHDA9 antibody and then detected by immunoblotting with anti‐TaHDA9 antibody, pan anti‐phospho‐S/T antibody (T91067M, Abmart), and pan‐anti‐Kac antibody (9681, Cell Signaling Technology).

### In vitro lysine deacetylation activity assays

The CDS fragments of TaRHC1A and TaHDA9 with Flag tags were each recombined into the expression vector His‐pCold‐TF. The recombinant His‐TF (pCold)‐TaRHC1A‐Flag, His‐TF‐TaHDA9‐Flag were expressed in *E. coli*. His‐TF‐TaRHC1A‐Flag protein was purified from *E. coli* cultured with the deacetylation inhibitors 5 mM TSA (HY‐15144, MedChemExpress) and 5 mM NAM (HY‐B0150, MedChemExpress) using a His‐tag Protein Purification Kit (P2226, Beyotime). His‐TF‐TaHDA9‐Flag protein was purified from *E. coli* cultured without the TSA and NAM using the His‐tag Protein Purification Kit (P2226, Beyotime). After cutting the His‐TF tag using thrombin (T8021, Solarbio), the above two recombinant proteins were used for in vitro lysine deacetylation.

For in vitro lysine deacetylation of TaRHC1A by TaHDA9, 10 μg purified TaRHC1A‐Flag and 10 μg TaHDA9‐Flag were incubated in a reaction buffer (50 mM Tris‐HCl, 50 mM NaCl, 6 mM MgCl2, 2 mM DTT, 1 mM ZnCl2; pH 8.0) at 30°C for 1 h. The samples were analyzed by SDS‐PAGE, followed by immunoblotting with anti‐Flag antibody (M20008) and pan‐anti‐Kac antibody (9681, Cell Signaling Technology).

### Site‐directed mutagenesis and expression

We performed site‐directed mutagenesis of position K222R (AAG‐AGG) and K11R (AAG‐AGG) within the His‐pCold‐TaRHC1A‐Flag plasmid using a Fast Site‐Directed Mutagenesis Kit (KM101, Tiangen), respectively. PCR was conducted using site‐specific primers (Table S16). Mutants were verified by DNA sequencing. His‐TF‐TaRHC1AK222R‐Flag, His‐TF‐TaRHC1AK11R‐Flag, and His‐TF‐TaRHC1A‐Flag were expressed in E. coli. The anti‐Flag antibody (M20008, Abmart) and pan‐anti‐Kac antibody (9681, Cell Signaling Technology) were used to detect the differential Kac levels of TaRHC1AK222R and TaRHC1A.

A 35S::TaRHC1AK222R‐Flag‐GFP vector was constructed using the His‐TF‐Flag TaRHC1AK222R plasmid as template. Following transient overexpression of recombinant 35S::TaRHC1AK222R‐Flag‐GFP and 35S::TaRHC1A‐Flag‐GFP in *N. benthamiana* leaf cells, the proteins were immunoprecipitated with anti‐Flag antibody (M20008, Abmart) and analyzed by immunoblotting with anti‐Flag antibody (M20008, Abmart) and pan‐anti‐Kac antibody (9681, Cell Signaling Technology).

### Antibody production

The full‐length RcPL3, TaHDA9, TaRHC1A, and TaRBOH3 protein sequences were used as antigens for generating polyclonal antibodies in rabbits by Abclonal (Wuhan, China), respectively. The specificity of these antibodies was detected by immunoblotting using wheat leaf proteins and protein purified by prokaryotic expression, respectively. The proteins of RcPL3‐OE, TaHDA9‐OE, TaRHC1A‐OE wheat lines and their controls were extracted to analyze their abundances in the RcPL3‐TaHDA9‐TaRHC1A‐TaRBOH3 model in wheat plants, by immunoblotting with using their specific antibodies, respectively. The anti‐actin antibody (A01050, Abbkine) was used as a loading control.

### Cell‐free assay

To detect whether TaRHC1A could affect the protein level of TaRBOH3, a cell‐free degradation assay was performed. Total proteins of wheat seedlings were extracted in degradation buffer (25 mM Tris‐HCl, pH 7.5, 10 mM NaCl, 10 mM MgCl2, 1 mM PMSF, 5 mM DTT, and 10 mM ATP) according to the method previously described [137] with minor modification. After centrifugation for 20 min at 14,000 × *g* and 4°C two times, cell debris was removed. The supernatant was collected, and the total protein extracts prepared from TaRHC1A‐OE and control (Fielder) seedlings were adjusted to equal concentration (+/−40 μM 26S proteasome inhibitor MG132) in the degradation buffer for each assay. For E. coli purified His‐Pcold‐TaRBOH3 protein degradation, 5 μg of recombinant His‐Pcold‐TaRBOH3 protein was incubated at 25°C with 0.5 mg wheat total proteins in degradation buffer for the individual assays with different time. The mixtures were detected by immunoblotting with anti‐His antibody (M20001, Abmart, China). The anti‐actin antibody (ABL1050, Abbkine) was used as a loading control. A cell‐free degradation assay was performed to detect the influence of TaHDA9 on TaRHC1A or TaRHC1AK222R in WT and TaHDA9‐OE lines.

The proteins of N. benthamiana leaves transiently expressing 35S::TaRHC1AK222R‐Flag‐GFP (+ /−10 μM 26S proteasome inhibitor MG132) were extracted and stayed at 25°C in degradation buffer for the individual assays with different time. The protein extractions were detected by immunoblotting with anti‐Flag antibody (M20008, Abmart). The anti‐actin antibody (A01050, Abbkine) was used as a loading control.

### H_2_O_2_ content and antioxidant enzyme activity detection

H_2_O_2_ contents were assessed by histochemical staining of wheat leaves by DAB, following the previous protocol [138]. For more accurate measurements, H_2_O_2_ levels were determined in triplicate using a Hydrogen Peroxide Assay Kit (S0038, Beyotime) or a Hydrogen Peroxide Content Assay Kit (BC3595, Solarbio) according to the manufacturer's instructions. Antioxidant enzyme activities of APX (BC0225, Solarbio, China), POD (BC0095, Solarbio), and CAT (BC0205, Solarbio) contents were measured in triplicate using commercial assay kits following kit instructions.

### Microscopic analysis of pathogen infection

To observe *Rc* infection in wheat plants, we sampled the stems of the transgenic wheat and their controls after *Rc* inoculation for 4 weeks, and then stained them with 2.5% glutaraldehyde. The *Rc* fungal was observed with an environmental scanning electron microscope (ESEM, Quanta45, FEI). A total of three infection stems were counted for each sample. Three independent biological replicates were performed.

To assess the cellular response of the transgenic wheat and wild type to *Rc* infection, cell necrotic death at the infection site was stained by trypan blue. The inoculated stems were detached from the plants and stained in a 0.5% solution of trypan blue in lactophenol for 2 days at room temperature and mounted in a solution of 2.5 mg/mL chloral hydrate. The stained segments were observed and photographed by a stereomicroscope (Olympus).

### Effect of HY‐19741 on wheat sharp eyespot resistance

To evaluate the antifungal activity of candidate small compounds that were selected by virtual screening, we identified the hyphal growth of *Rc* in potato dextrose agar (PDA) medium treated with 50, 100, and 150 μM small compounds, including HY‐19741 (CAS:1430844‐80‐6), HY‐13258 (CAS:2245892‐85‐5), HY‐102013 (CAS:1912399‐75‐7), HY‐12793 (CAS:1643913‐93‐2), and HY‐136895 (CAS:945396‐55‐4), respectively. The treatment with the dimethylsulfoxide (DMSO) was used as a control. From 2 to 5 dpi, we scanned the PDA plates and recorded the area of the mycelial colony with ImageJ software. The mycelium growth inhibition rate was evaluated by measuring the mycelial colony diameter using the following equation [139].

I%=[(C−d)−(T−d)]/(C−d)×100,

where *C* represents the diameter of the control colony, *d* is the diameter of the original mycelial disk (5 mm), and *T* is the diameter of the colony treated with HY‐19741 or other compounds.

The boiled and sterilized wheat kernels were treated with 50 μM HY‐19741 or DMSO (0.05%) for 10 min, respectively. Then, five pieces of activated medium with *Rc* mycelia were transferred into each treatment with HY‐19741 or DMSO for inoculum preparation. The evaluation of sharp eyespot resistance in the greenhouse according to the method previously described [28]. After *Rc* inoculation for 4 weeks, disease phenotype of wheat seedlings was evaluated, with DI calculated as above. Twelve infection stems were counted for each experiment. At least three independent biological replicates were performed.

### Quantification and statistical analysis

Statistical analysis for two‐group comparisons was performed using the two‐tailed Student's *t*‐test. For datasets containing three or more groups, one‐way ANOVA was conducted, followed by post hoc multiple comparisons in IBM SPSS Statistics 26. Dunnett's test was used to compare each experimental group versus the control group, whereas Tukey's test was applied for all pairwise comparisons among groups. Identical significance thresholds were applied across all statistical tests: *p* < 0.05 (*), *p* < 0.01 (**), *p* < 0.001 (***), and *p* < 0.0001 (****).

## AUTHOR CONTRIBUTIONS

**Yan Ren**: Investigation; conceptualization; writing—original draft; writing—review and editing; data curation; validation; methodology; funding acquisition; formal analysis; visualization; resources. **Ti Liu**: Methodology; data curation; formal analysis; validation; investigation. **Heying Zhao**: Investigation; validation; formal analysis; data curation. **Qirun Qian**: Investigation; validation. **Lufei Cui**: Investigation; validation. **Zhipeng Sun**: Investigation; validation; formal analysis. **Wenkai Yang**: Investigation; validation. **Peng Guo**: Investigation; validation. **Sisheng Wang**: Investigation; validation. **Lingran Zhang**: Validation; formal analysis. **Congwei Sun**: Formal analysis; data curation; software. **Aolin Jia**: Formal analysis. **Ning Zhang**: Conceptualization; validation; formal analysis; supervision. **Feng Chen**: Conceptualization; supervision; writing—original draft; writing—review and editing; project administration; resources.

## CONFLICT OF INTEREST STATEMENT

The authors declare no conflicts of interest.

## ETHIC STATEMENT

No animals or humans were involved in this study.

## Supporting information


**Figure S1:** Fluorescence microscopy, genomic annotation, and variants of *Rc*.
**Figure S2:** Genome collinearity of *Rc* with *Rc* (A and B), *Rc* with *R. solani* (C and D), *Rc* with *B. botryosum* (E and F), *Rc* with *M. roreri* (G and H), *Rc* with *P. placenta* (I and J), and *Rc* with *U. maydis* (K), respectively.
**Figure S3:** Venn diagram showing core and dispensable gene families of effector genes from five *Rc* isolates.
**Figure S4:** GO (Gene Ontology) (A−C) and KEGG (Kyoto Encyclopedia of Genes and Genomes) enrichment analysis (D) of identified upregulated genes at least one time point during *Rc* infection.
**Figure S5:** Pathogenicity distribution and genome‑wide association study (GWAS) analysis of the 207 *Rc* isolates.
**Figure S6:** The feasibility of VIGS system in verifying the role of effector.
**Figure S7:** Functional verification by *RcPL3*‐overexpression lines in wheat.
**Figure S8:** The tissue‐specific expressions of three genes (*TaHDA9*, *TaRHC1A*, and *TaRBOH3*), interaction of TaHDA9 and RcPL3, the specificity for the anti‐TaHDA9 antibody, and phylogenetic tree of HDAC family.
**Figure S9:** The R267 in TaHDA9 is a key site to interact with RcPL3.
**Figure S10:** Functional verification by VIGS, EMS mutants, and CRISPR‐Cas9 gene editing lines for *TaHDA9* and by overexpression for *TaHDA9* ^*R267A*^.
**Figure S11:** K222 in TaRHC1A is a crucial site de‐acetylated by TaHDA9, degradation of TaRHC1A through K222 de‐acetylated by TaHDA9, and functional verification of TaRHC1A by VIGS and EMS mutants.
**Figure S12:** Volcano plot of differentially expressed genes and enzyme activities of ROS‐related proteins in *TaRHC1A*‑OE plants.
**Figure S13:** Prediction of the interaction between TaRHC1A and TaRBOH3, and the ubiquitination and degradation of TaHDA9 by TaRHC1A.
**Figure S14:** Yeast two‐hybrid assay shows no interaction between RcPL3 and TaRHC1A.
**Figure S15:** An agar diffusion assay of *Rc* in a potato dextrose agar (PDA) medium was performed using the five compounds.
**Figure S16:** Immunoblots detect the protein abundance of endogenous TaHDA9 (A), TaRBOH3 (B), and TaRHC1A (C) in wheat plants with or without treatment of 50 μM exogenous HY‐19741 and the expression levels of four ROS‐related genes (*TaPOD1*, *TaPOD3*, *TaSOD1*, and *TaAPX2*) with or without treatment of 50 μM exogenous HY‐19741 (D−G).

**Table S1:** Pathogenicity data and ITS sequence information for 267 *Rc* isolates in this study and binucleate *Rhizoctonia* representative isolates for the phylogenetic tree.
**Table S2:** Comparison of *Rc* disease index among the four cultivars in the tested 267 *Rc* isolates.
**Table S3:** Genome assembly and annotation of five selected *Rc* isolates.
**Table S4:** Classification and annotation of repetitive sequences, BUSCO evaluation of genome completeness, and protein‐coding genes and gene families in the five *Rc* genomes.
**Table S5:** Summary of *Rc*‐specific gene families and genes.
**Table S6:** Protein family database (Pfam) annotation of *Rc‐*expanded gene families and effector proteins.
**Table S7:** Pfam annotation of core gene families (CGF) and dispensable gene families (DGF) of *Rc* effectors.
**Table S8:** Upregulated genes at least one time point during *Rc* infection.
**Table S9:** Prediction of conserved domain for 43 upregulated effectors in R0301.
**Table S10:** Repetitive SNPs and significance revealed by genome‐wide association study in at least two groups of pathogenicity phenotypes.
**Table S11:** Genome‐wide association study (GWAS) results of 43 upregulated effectors.
**Table S12:** The haplotype analysis of *RcPL3* and *TaHDA9*.
**Table S13:** Quantitative values of the proteins in all figures and supplementary figures.
**Table S14:** The screened 267 compounds were identified after three rounds of molecular docking.
**Table S15:** BlastP analysis of protein sequence similarities between RcPL3 and homologous proteins.
**Table S16:** The primers used in this study.

## Data Availability

The data that support the findings of this study are available in the supplementary material of this article. The de novo sequencing, resequencing, and transcriptome data generated during this study have been deposited in the NCBI Sequence Read Archive with the Bio Project accession numbers PRJNA1199898 (https://ncbi.nlm.nih.gov/bioproject/PRJNA1199898), PRJNA1238469 (https://ncbi.nlm.nih.gov/bioproject/PRJNA1238469), and PRJNA1063871 (https://ncbi.nlm.nih.gov/bioproject/PRJNA1063871). The data and scripts used are saved in GitHub https://github.com/20260820ren/Ren2026iMeta. Supplementary materials (figures, tables, graphical abstract, slides, videos, Chinese translated version, and updated materials) may be found in the online DOI or iMeta Science http://www.imeta.science/.
