# Supplementary material for: Infection mechanisms of Rhizoctonia cerealis in wheat
Source: Imeta. 2026 Oct 5;5(5):e70174. doi: 10.1002/imt2.70174 (PMC13638802; doi:10.1002/imt2.70174)
Supplement: Supplementary file 1 [file IMT2-5-e70174-s002.docx]

Supporting Information to

**Infection mechanisms of *Rhizoctonia cerealis* in wheat**

**Running title:** Infection mechanisms of *Rhizoctonia cerealis* in wheat

Yan Ren^#^, Ti Liu^#^, Heying Zhao, Qirun Qian, Lufei Cui, Zhipeng Sun, Wenkai Yang, Peng Guo, Sisheng Wang, Lingran Zhang, Congwei Sun, Aolin Jia, Ning Zhang^*^, Feng Chen^*^

State Key Laboratory of High-Efficiency Production of Wheat-Maize Double Cropping, Agronomy College, Henan Agricultural University, Zhengzhou 450046, China

^#^These authors contributed equally: Yan Ren, Ti Liu

^*^Correspondence: fengchen@henau.edu.cn (Feng Chen), zhangning88012@126.com (Ning Zhang)

**SUPPLEMENTARY FIGURES**

**Figure S1 Fluorescence microscope, genomic annotation, and variants of *R. cerealis* (*Rc*).** (A) Fluorescence microscope confirmed two nuclei per cell in *Rc* isolates. Arrows point to nucleus (N) and septa (S). Scale bars, 20 μm. (B) Neighbor-joining tree based on rDNA-ITS sequences of 267 *Rc* isolates from this study and binucleate *Rhizoctonia* isolates from 21 anastomosis groups. Blue highlights represent isolates of 17 anastomosis groups (AGs); red highlights represent 168 isolates with a single ITS sequence; yellow highlights represent 99 isolates with multiple ITS sequences. (C) Statistical plot of the number of predicted genes supported by three prediction methods in five *Rc* isolates. R0301 (a), R5-3 (b), R1-8 (c), R10-1 (d), and R1-6 (e). Three predictions methods included ab initio predictions (Denovo), protein homology (Homolog), and RNA-seq read mapping (RNA-seq). (D−G) Characterization of the pan-genome in *Rc*. Pfam annotation of genes in core gene families (D), genes in dispensable gene families (E), number of added gene families identified from increased isolates (F), and pan-genome and core genome (G). (H) Phylogenetic analyses of genomic variants in *Rc*. AH, Anhui Province; HB, Hubei Province; JS, Jiangsu Province; SC, Sichuan Province; SD, Shandong Province; SX, Shaanxi Province; ITS, internal transcribed spacer.

**Figure S2** **Genome collinearity of *Rc* with *Rc* (A and B), *Rc* with *R. solani* (C and D), *Rc* with *B. botryosum* (E and F), *Rc* with *M. roreri* (G and H), *Rc* with *P. placenta* (I and J), and *Rc* with *U. maydis* (K), respectively.**

**Figure S3** **Venn diagram showing core and dispensable gene families of effector genes from five *Rc* isolates.**

**Figure S4 GO (Gene Ontology) (A−C) and KEGG (Kyoto Encyclopedia of Genes and Genomes) enrichment analysis (D) of identified upregulated genes** **at least one time point during *Rc*** **infection.**

**Figure S5 Pathogenicity distribution and** **genome‑wide association study (GWAS) analysis of the 207 *Rc* isolates.** (A−G) Frequency distributions of average sharp eyespot disease index (DI) for *Rc* isolates across seven pathogenicity phenotypes (*n* = 3). The average DI in Chinese Spring (A); the average DI in Yumai 49 (B); the average DI in Aikang 58 (C); the average DI in Zhoumai 18 (D); the average DI of all four cultivars (E); the average DI of the Chinese spring and Yumai 49 (F); the average DI in the AK58 and Zhoumai18 (G). Q-Q plots (H−M) and Manhattan (N−S) for *Rc* pathogenicity. CS, Chinese Spring; YM49, Yumai 49; AK58, Aikang 58; ZM18, Zhoumai 18; CS+YM49+AK58+ZM18 (average DI of all four cultivars); CS+YM49 (average DI of these two cultivars); and AK58+ZM18 (average DI of these two cultivars). (T) Linkage disequilibrium (LD) decay for *Rc* populations from eight Chinese provinces. AH indicates Anhui; HB indicates Hubei; HN indicates Henan; JS indicates Jiangsu; SC indicates Sichuan; SD indicates Shandong; SX indicates Shaanxi; ZJ indicates Zhejiang. (U) The statistical analysis of cell death phenotype of *N. benthamiana* leaves across eight biological replicates. Data in (U) are presented as the mean values ± SE with individual data point (*****p* < 0.0001; one-way ANOVA with Dunnett’s multiple comparisons test).

**Figure S6** **The feasibility of VIGS system in verifying the role of effector.** (A−C) Functional verification of *RcOSP1* by virus-induced gene silencing (VIGS). Sharp eyespot responses (A), relative expression levels (B), and average disease index (C) of wild type (WT), BSMV:00, and BSMV:RcOSP1 plants inoculated with *Rc* isolate R0301. The red dotted box shows the sites of the SE infection. (D−G) Malondialdehyde (MDA) contents and enzyme activities of superoxide dismutase (SOD), catalase (CAT), and peroxidase (POD) in WT, BSMV:00, and BMSV:RcPL3 wheat plants. Scale bars, 1 cm in (A). Data in (B and C) are presented as the mean values ± SE with individual data point (**p* < 0.05 and ***p* < 0.01; unpaired two-sided *t*-test with at least three biologically independent replicates). Data in (D−G) are presented as the mean values ± SE with individual data point (**p* < 0.05, ***p* < 0.01, ****p* < 0.001 and *****p* < 0.0001; one-way ANOVA with Dunnett’s multiple comparisons test).

**Figure S7 Functional verification by *RcPL3*-overexpression** **lines in wheat.** (A) Stems of T_2_ generation *RcPL3*-OE lines at 22 dpi with R0301 showed increased disease development. (B) Immunoblots of RcPL3 signal protein in the wild type (WT) and *RcPL3-*overexpression (*OE*) lines. CBB, Coomassie brilliant blue. Average disease index (C) (12 plants per replicate, *n* = 6), trypan blue staining of necrotic cell death and mycelial coverage (D), *Rc* biomass (E), and H_2_O_2_ content (F) of the WT and *RcPL3*-OE lines. Scale bars, 1 cm in (A) and 100 μm in (D). Red dotted box in (A) shows the infected sites by *Rc*. Red arrows in (D) indicate *Rc* hypha. Data in (C), and (E and F) are presented as the mean values ±SE with individual data point (***p* < 0.01, ****p* < 0.001 and *****p* < 0.0001; one-way ANOVA with Dunnett’s multiple comparisons test).

**Figure S8 The tissue-specific expressions of three genes (*TaHDA9*, *TaRHC1A*, and *TaRBOH3*), interaction of TaHDA9 and RcPL3, the specificity for the anti-TaHDA9 antibody, and phylogenetic tree of HDAC family.** (A) The tissue-specific expressions of *TaHDA9*, *TaRHC1A*, and *TaRBOH3* in wheat from WheatPro (https://www.csuligroup.com/WheatPro/). The basal BS_BPST, booting stage_basal part of stem; HS_BPST, heading stage_basal part of stem; FS_BPST, filling stage_ basal part of stem. (B) Interaction of TaHDA9 with RcPL3_HapA and RcPL3_HapB by yeast two-hybrid. (C) The specificity of the anti-TaHDA9 antibody was detected by immunoblotting using TaHDA9 protein purified by prokaryotic expression and wheat leaf proteins. (D) Phylogenetic tree and sequence similarity analysis of HDAC family proteins in wheat.

 **Figure S9 The R267 in TaHDA9 is a key site to interact with RcPL3.** (A) Verification of the generated mutant sites in *TaHDA9^E94A^*, *TaHDA9^D199A^*, *TaHDA9^D266A^*, *TaHDA9^R267A^*, *TaHDA9^L268A^*, and *TaHDA9^D266A/R267A/L268A^* by Sanger sequencing*.* E94A, GAG-GCG; D199A, GAC-GCC; D266A, GAC-GCC; R267A, CGC-GCC; L268A, CTA-GCA. (B−E) Pull-down assays of RcPL3 with TaHDA9 or six TaHDA9 mutants to confirm the key interaction sites between RcPL3 and TaHDA9. (F−I) Quantitative values of the proteins in Figure S9B−E. Data in (F and I) are presented as the mean values ±SE with individual data point (**p* < 0.05, ****p* < 0.001 and *****p* < 0.0001; one-way ANOVA with Dunnett’s multiple comparisons test). Data in (G and H) are presented as the mean values ± SE with individual data point (**p* < 0.05; unpaired two-sided *t*-test with at least three biologically independent replicates).

**Figure S10** **Functional verification by VIGS, EMS mutants and CRISPR–Cas9 gene editing lines for *TaHDA9* and by overexpression for *TaHDA9****^R267A^***.** (A−C) Functional verification of *TaHDA9* by VIGS. Phenotypes for VIGS-treated plants (A), relative expression levels (B), and average disease index of wild type (WT) (C) (12 plants per replicate, *n* = 3), BSMV:00 and BMSV: TaHDA9 plants. The red dotted box shows the sites of the SE infection. (D−H) EMS-mutagenized tetraploid wheat Kronos is used to verify the function of *TaHDA9*. Mutation sites (D), SE responses (E), DAB (3,3’-diamino benzidine hydrochloride) staining (F), SE disease index (G) (12 plants per replicate, *n* = 3), and H_2_O_2_ contents (H) in WT and mutant plants. (I−N) *TaHDA*9-Cas9 lines significantly enhance SE resistance. Comparison of disease response (I), Sanger sequencing results of *TaHDA9*-edited lines and the editing sites in *TaHDA9*-edited lines (J), trypan blue staining of necrotic cell death and mycelial coverage (K) and SE disease index (L) (12 plants per replicate, *n* = 3), H_2_O_2_ content (M) and *Rc* biomass (N) in WT and *TaHDA9*-edited lines. (O−S) Genetic transformation revealed that *TaHDA9^R267A^* positively modulated SE resistance in wheat. Comparison of disease response (O), the relative expression levels (P), SE disease index (Q), *Rc* biomass (R) and H_2_O_2_ content (S) in *TaHDA9^R267A^*-OE lines and their negative control. Scale bars, 1 cm in (A, E, I, and O) and 100 μm in (K). Data in (B, and Q−S) are presented as the mean ±SE with individual data point (****p* < 0.001; unpaired two-sided *t*-test with at least three biologically independent replicates). Data in (C), (G and H) and (L−N) are presented as the mean values ±SE with individual data point (**p* < 0.05, ***p* < 0.01, ****p* < 0.001 and *****p* < 0.0001; one-way ANOVA with Dunnett’s multiple comparisons test).

**Figure S11 K222 in TaRHC1A is a crucial site de-acetylated by TaHDA9,** **degradation of TaRHC1A through K222 de-acetylated by TaHDA9, and functional verification of *TaRHC1A* by VIGS and EMS mutants.** (A) Raw mass spectrum of TaRHC1A protein produced from *E. coli*. (B and C) Verification of the generated mutant sites in TaRHC1A^K222R^ and TaRHC1A^K11R^ by Sanger sequencing. K222R, AAG-AGG; K11R, AAG-AGG. (D) Immunoblots detect no significant change of acetylation level of TaRHC1A^K11R^ in *E. coli.* (E and F) *TaHDA9*-OE plants had lower TaRHC1A protein level by the cell-free system. Recombinant His-pCold-TaRHC1A protein is added to the total protein extracted from seedlings of WT Fielder (E) and *TaHDA9*-OE plants (F). (G and H) Both *TaHDA9*-OE and Fielder plants show a lower level of TaRHC1A^K222R^ protein by the cell-free system. (I) Immunoblots detect transient expression of Ubi::TaRHC1A-GFP and Ubi::TaRHC1A^K222R^-GFP in *TaRHC1A*-OE (left) and Fielder (right) protoplasts of wheat. (J) The protein level of TaRHC1A^K222R^ protein is significantly decreased in wheat and tobacco possibly through the 26S proteasome pathway. Recombinant His-pCold-TaRHC1A^K222R^ is added to the total protein from WT plants with or without 26S proteasome inhibitor MG132. The proteins of *N. benthamiana* leaves transiently expressing 35S::TaRHC1A^K222R^-Flag-GFP with or without MG132 was extracted. (K−M). Functional verification of *TaRHC1A* by the barley strip mosaic virus (BSMV)-mediated VIGS. Phenotypes for BSMV-infected plants (K), relative expression level (L), and average disease index (M) (12 plants per replicate, *n* = 5) of wild type (WT), BSMV:00 and BMSV: TaRHC1A plants. (N−Q) EMS mutants with premature stop codon of *TaRHC1A* in Kronos showed decreased SE resistance. Mutation site in *TaRHC1A* is shown by the red box (O). Phenotypes for BSMV-infected plants, SE disease index, and H_2_O_2_ contents in WT and *TaRHC1A* mutants (N, P, and Q). Scale bars, 1 cm in (K and N). Data in (L) and (P and Q) are presented as the mean ± SE with individual data point (***p* < 0.01 and *****p* < 0.0001; unpaired two-sided *t*-test with at least three biologically independent replicates). Data in (M) are presented as the mean values ±SE with individual data point (***p* < 0.01; one-way ANOVA with Dunnett’s multiple comparisons test). In (D−J), the experiments were conducted with at least three biological replicates showing similar results.

**Figure S12** **Volcano plot of differentially expressed genes and enzyme activities of ROS-related proteins in *TaRHC1A*‑OE plants.** (A) Volcano plot for differentially expressed gene (DEGs) number between *TaRHC1A*-OE plants and Fielder based on transcriptome sequencing. Up, up-regulated; Down, down-regulated. (B and C) Enzyme activities for APX (B) and POD (C) contents in Fielder and *TaRHC1A*-OE plants. Data in (B and C) are presented as the mean ± SE with individual data point (****p* < 0.001; unpaired two-sided *t*-test with at least three biologically independent replicates).

**Figure S13 Prediction of the interaction between TaRHC1A and TaRBOH3, and the ubiquitination and degradation of TaHDA9 by TaRHC1A.** (A) The interaction prediction of TaRHC1A and TaRBOH3 by Alphafold2 analysis. (B) Immunoblots detect that the protein abundance of endogenous TaRBOH3 is decreased in *TaRHC1A*-OE plants. (C) H_2_O_2_ detection by DAB staining of BSMV:00 and BSMV:TaRBOH3 plants. (D and E) TaRHC1A reduces the accumulation of TaHDA9 protein. The protein level of TaHDA9 is significantly decreased in *TaRHC1A*-OE plants possibly through the 26S proteasome pathway using the cell-free system (D). Immunoblots detect transient expression of 35S::TaRHC1A-His-GFP with (+) or without (−) 35S::TaHDA9-Flag-GFP in *N. benthamiana* (E). In (D and E), the experiments were conducted with three biological replicates showing similar results. Scale bars, 200 μm in (C).

**Figure S14 Yeast two-hybrid assay shows no interaction between RcPL3 and TaRHC1A.**

**Figure S15 An agar diffusion assay of *Rc* in a potato dextrose agar (PDA) medium was performed using the five compounds**. (A and C), Images of *Rc* treated with 100 μM and 150 μM of different compounds. (B and D), Analysis of variance of mycelium growth areas under different treatment concentrations of 100 μM and 150 μM. The dimethylsulfoxide (DMSO) was used as a control. Scale bars, 1 cm in (A and C). Data in (B and D) are presented as the mean ± SE with individual data point. Different letters indicate significant differences (*p* < 0.05), as determined by one-way analysis of variance (ANOVA) with Tukey’ multiple comparisons test.

**Figure S16** **Immunoblots detect the protein abundance of endogenous TaHDA9 (A), TaRBOH3 (B), and TaRHC1A (C) in wheat plants with or without treatment of 50 μM exogenous HY-19741 and the expression levels of four ROS-related genes (*TaPOD1*, *TaPOD3*, *TaSOD1*, and *TaAPX2*) with or without treatment of 50 μM exogenous HY-19741 (D−G).** Data in (D−G) are presented as the mean ± SE with individual data point (****p* < 0.001 and *****p* < 0.0001; Unpaired two-sided *t*-test with at least three biologically independent replicates).
